# In vitro reconstitution of vertebrate Sonic Hedgehog protein cholesterolysis

**DOI:** 10.64898/2026.03.09.710561

**Published:** 2026-03-11

**Authors:** Dayton C. Seidel, Andrew G. Wagner, John L. Pezzullo, Katherine A. Thayer, Seth Beadle, Margot L. Olejarczyk, José-Luis Giner, Brian P. Callahan

**Affiliations:** 1 Department of Chemistry, Binghamton University, State University of New York, Binghamton, New York 13902, United States; 2 Department of Chemistry, State University of New York College of Environmental Science and Forestry, Syracuse, New York 13210, United States

## Abstract

Extracellular secretion of the oncogenic sonic hedgehog signaling ligand is contingent on its release from a precursor protein through peptide bond cholesterolysis, mediated by the hedgehog C-terminal domain, SHhC. In this work, we describe the in vitro reconstitution of cholesterolysis activity for SHhC domains from vertebrate model organisms, *Xenopus laevis* (Xla) and *Danio rerio* (Dre). Cholesterolysis is assayed continuously in multi-well plates by monitoring changes in fluorescence resonance energy transfer (FRET) from an engineered precursor construct, expressed in *E. coli* and purified in soluble form. Using this FRET assay, we found that Xla and Dre SHhC exhibit high substrate stereospecificity, accepting cholesterol, (K_M_, 1–2 μM, cholesterolysis t_1/2_ of ~11 min) while rejecting the 3-alpha epimer, epi-cholesterol (K_M_ > 100 μM, t_1/2_ > 10 hr). By screening a 96-member detergent/surfactant library for compatibility with SHhC activity, we identify cationic detergents that inhibit cholesterolysis and find a shared preference for the zwitterionic n-dodecyl-phosphocholine (DPC, Fos-choline-12), which supported the fastest reaction kinetics. Lastly, we report that alanine point mutation at a conserved aspartate residue (D46A) in Xla SHhC and Dre SHhC blocks cholesterolysis; however, activity could be chemically rescued with rationally designed hyper-nucleophilic sterols. Of those sterols, 2-beta carboxy cholestanol was active as a substrate with D46A variants only; the remaining sterols were accepted by both D46A and wild-type SHhC. In summary, we have established the first in vitro kinetic assay to continuously monitor enzymatic activity of wild-type and mutant vertebrate SHhC domains in multi-well plates, a key step toward pharmacological manipulation of Sonic hedgehog protein biosynthesis in vivo.

## INTRODUCTION:

The extracellular Sonic hedgehog ligand (SHhN) initiates vital cell/cell signaling in pre-natal development through adulthood.^1^ Congenital mutations that diminish Sonic hedgehog protein expression are associated with holoprosencephaly, a potentially fatal brain disorder,^2, 3^ while chronic, overexpression appears to promote the growth of certain tumors. ^4–6^

**Scheme 1.**
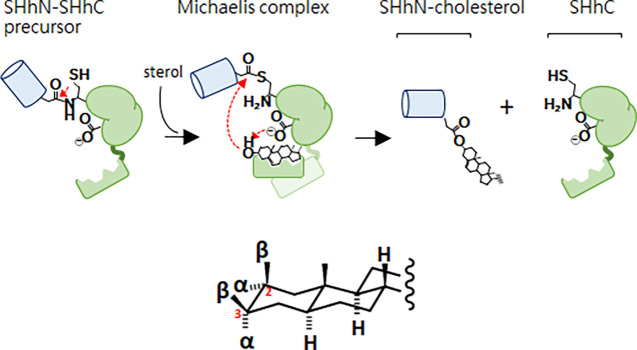
(top) Sonic hedgehog precursor protein (SHhN-SHhC) undergoes a specialized form of autoprocessing called peptide bond cholesterolysis, brought about by the precursor’s enzymatic SHhC domain. A conserved cysteine residue (Cys1) of SHhC initiates the transformation by rearranging the backbone peptide bond at the SHhN-SHhC junction, forming an internal thioester; this SHhN~SHhC thioester is then displaced by substrate cholesterol, facilitated through general base catalysis involving a conserved aspartate residue (D46). (bottom) Cholestane stereochemistry and numbering system relevant to the present work.

Human SHhN and homologous proteins of metazoans are expressed as a bifunctional precursor with the hedgehog ligand flanked by a C-terminal cholesterolysis domain, SHhC. ^7–11^ Before SHhN ligand can be secreted extracellularly for biological signaling, SHhC must cleave itself from SHhN and covalently join the ligand’s newly formed carboxy terminus to a molecule of cholesterol (Scheme 1).^12, 13^ This unusual cleavage/sterylation event occurs site-specifically at a conserved Gly(−1)↓Cys(1) motif that separates SHhN from SHhC.^14, 15^ Residues are numbered here relative to SHhC, where the first amino acid of SHhC is 1 and the last residue of SHhN is −1.

### SCHEME 1

As depicted above, cholesterolysis begins with isomerization of the Gly(−1)-Cys(1) backbone peptide bond, activating the Gly(−1) as an internal thioester. Following substrate cholesterol binding, the lipid’s 3-β hydroxyl group is subjected to general base catalysis by a conserved aspartate residue (D46) of SHhC. Displacement of the internal thioester by cholesterol completes the chemical transformation.^1^ No cofactors/coenzymes are required. Cholesterolysis appears to be single turnover in the cell and entirely self-catalyzed by SHhC.^7, 14, 15^ The SHhN ligand adjacent to the cleavage site is a bystander; its replacement with unrelated polypeptides leaves SHhC activity intact in vitro and in vivo.^16–20^ Mutations that disable SHhC^21^ or extreme depletion of endogenous cholesterol,^22^ result in retention of unprocessed SHhN-SHhC in the secretory pathway where the precursor is targeted for degradation by the proteasome, blocking downstream signaling.^23, 24^

Biochemical studies, mutational analysis and small molecule discovery efforts on hedgehog cholesterolysis have focused on the *Drosophila melanogaster* domain, Dme HhC. ^17, 25–29^ Initial biochemical evidence for cholesterolysis was collected using this purified domain.^15^ Dme HhC is reliably overexpressed in *E. coli* in soluble, cholesterolysis active form and tolerates N- and C-terminal fusions to heterologous proteins and tag sequences.^17^ To date, Dme HhC remains the only cholesterolysis domain with experimentally determined high resolution structure data,^14, 30, 31^ although this information is limited to the protein’s first ~150 amino acids; the final ~80 amino acids of Dme HhC, which are necessary for recognition of substrate cholesterol, have so far resisted structural analysis.

*Drosophila* HhC and human SHhC share only 32% amino acid identity (45% similarity). Unlike Dme HhC, the native folding of human SHhC appears to require protein disulfide isomerase^23^ and asparagine glycosylation,^32^ features that may explain challenges in the heterologous expression of functional human SHhC in *E. coli*. It also seems worth noting that *Drosophila melanogaster* is a sterol auxotroph,^33^ a characteristic that could broaden Dme HhC substrate tolerance for (scavenged) sterols relative to human SHhC. Indeed, the native substrate for insect HhC domains is not known and may be a fungal or phytosterol.

Identifying SHhC cholesterolysis domains that are experimentally tractable and more closely related to human SHhC remains an important goal. In this work, we pursue SHhC domains from the vertebrate model organisms, *Xenopus laevis* (African clawed frog) and *Danio rerio* (Zebrafish). Pairwise comparisons of *Xenopus laevis* and *Danio rerio* SHhC domains with human SHhC show 49% identity/59% similarity and 46% identity/60% similarity, respectively (Supporting Table 1, Supporting Figure 1). Similar to human SHhN, the mature Zebrafish and Xenopus SHhN ligands are derived from SHhN-SHhC precursor cholesterolysis and regulate complex multicellular behavior. The zebrafish SHhN ligand participates in retinal development^34^, tooth formation^35^, and neurogenesis^36^. Signaling by *Xenopus laevis* SHhN is implicated in organogenesis^37, 38^ and tissue regeneration^39^.

As a step toward pharmacological manipulation of sonic hedgehog protein biogenesis, we establish here the first continuous, in vitro kinetic assays to monitor *Xenopus laevis* and *Danio rerio* SHhC cholesterolysis. Assays are conducted in multi-well plates with an engineered FRET-active SHhC reporter produced in soluble form using *E. coli*. Compared to conventional gel-based SHhC activity assays, the FRET-method improves the speed of sample throughput and provides a more complete picture of reaction progress through realtime data acquisition. We use this method to show that vertebrate SHhC domains, like their invertebrate counterpart Dme HhC, exhibit low micromolar affinity for substrate cholesterol while rejecting epi-cholesterol, the 3-epimer of cholesterol. We observed that both vertebrate SHhC domains are susceptible to precursor thiolysis in the absence of cholesterol, suggesting that internal thioester formation and substrate binding by SHhC are not strictly coupled. Screening a 96-member library of potential sterol solubilizing agents for in vitro SHhC cholesterolysis, we found that vertebrate and invertebrate cholesterolysis domains displayed a shared preference for the phosphatidylcholine analogue, fos-choline 12 (DPC). Lastly, we report that alanine point mutation at a conserved aspartate residue (D46) in the zebrafish and Xenopus SHhC domains allows for bioorthogonal “chemical rescue”, where SHhC cleavage/sterylation activity is dependent on exogenous hyper-nucleophilic sterol.

## RESULTS AND DISCUSSION:

### Heterologous *E. coli* expression of vertebrate SHhC reporter constructs

To explore the viability of *in vitro* enzymatic studies involving *Xenopus laevis* and *Danio rerio* SHhC domains, hereafter Xla SHhC and Dre SHhC, we cloned codon optimized fragments encompassing the respective proteins along with a short flanking SHhN peptide into an arabinose-inducible FRET reporter expression plasmid, extending the approach we used previously for *Drosophila melanogaster* HhC (Dme HhC).^17^ The encoded polyprotein, C-H-Y, has an N-terminal cyan fluorescent protein (C), followed by the hedgehog SHhC domain (H), fused to yellow fluorescent protein (Y) with a C-terminal His6 purification tag (Supporting Figure 2).

Along with wild-type FRET reporter constructs, we prepared two alanine point mutants of Xla SHhC and Dre SHhC: a C1A mutant to serve as a negative control where the catalytically essential nucleophilic thiol of SHhC is removed; and a D46A mutant, where the putative general base function of the D46 side-chain carboxyl group is eliminated. The D46A mutation in *Drosophila* HhC and human SHhC have proven amenable to chemical rescue of their cleavage/sterylation activity.^16, 40^

FRET reporter constructs of wild-type and the two point mutants of Xla and Dre SHhC were overexpressed in *E. coli* and purified in soluble form using standard Ni-NTA chromatography (Supporting Figure 3). Compared with Dme HhC, yields of purified C-H-Y incorporating the vertebrate domains were reduced by a factor of 4.5 and 3 for Xla SHhC and Dre SHhC respectively. Two different E. coli strains were tested, LMG194 and AI-BL21; yields were slightly better in AI-BL21. Analysis of elution fractions by SDS-PAGE indicated that spontaneous, hydrolytic autoprocessing of the Xla and Dre SHhC constructs was greater than with Dme HhC. Despite reduced yields with the vertebrate domains, the WT, C1A and D46A SHhC constructs exhibited FRET signal five to seven-fold above background and displayed the expected cholesterolysis activity, inactivity, or conditional activity, as described below.

### Vertebrate hedgehog cholesterolysis activity in vitro by continuous FRET assay

Xla and Dre SHhC cholesterolysis have been examined before using label-based, endpoint approaches. Xla SHhC activity was detected in the presence of Xenopus egg extracts with in vitro translated ^35^S-labeled Xenopus Sonic Hh precursor protein.^23^ A nonradioactive chemical tagging approach involving an alkynyl-modified cholesterol, click chemistry and in-gel fluorescence, was developed for Dre SHhC cholesterolysis. ^41^

**Figure 1.**
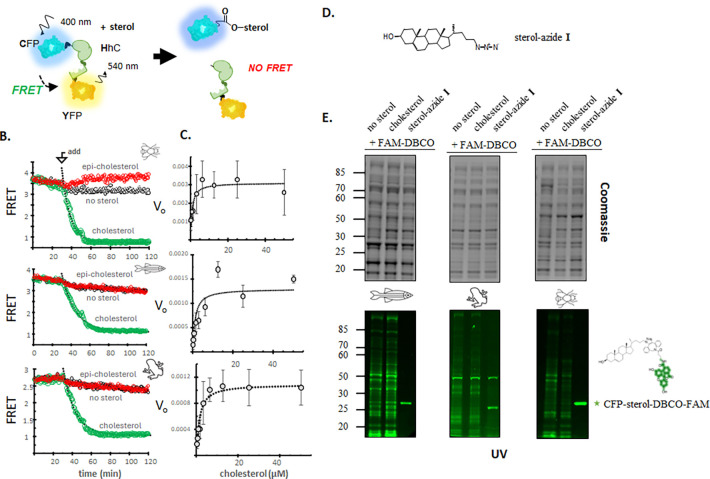
Continuous in vitro assay of vertebrate SHhC cholesterolysis activity. **A.** FRET-active cholesterolysis reporter. **B.** Kinetic assays with substrate cholesterol and inactive epicholesterol. After a thirty-minute incubation of samples containing C-H-Y reporter, sterol was added from an ethanol stock to final concentration of 50 μM, 2% ethanol. “No sterol” control reactions had the same volume of ethanol added to evaluate possible solvent effects. Representative kinetic traces are shown for wild-type C-H-Y reporters for *Drosophila melanogaster* (Dme), *Danio rerio* (Dre) and *Xenopus laevis* (Xla). **C.** Concentration-response plots for cholesterolysis. Wild-type C-H-Y reporter constructs with Dme, Dre and Xla domains exhibited Michaelis-Menten type behavior in plots of initial rate of FRET-loss as a function of increasing initial cholesterol concentration. Derived K_M_ values were ~ 1 μM. **D**. Structure of clickable cholesterol azide analog. **E**. Covalent sterylation. Clickable cholesterol (I) was used as an alternative substrate for wild-type C-H-Y reporter constructs of Dme, Dre and Xla in the presence of E. coli soluble lysate. Completed reactions were mixed with DBCO-FAM, 1:1 with (**I**), separated by denaturing SDS-PAGE and imaged for fluorescence using BioRad EZ Gel Doc. Negative control reactions without (I), showed an unspecific laddering of the fluorescent label (lanes 1–2). Samples with clickable sterol showed a prominent band at the expected molecular weight for sterylated CFP (lane 3).

For higher throughput and continuous monitoring of reaction progress, we sought to assay Xla and Dre SHhC cholesterolysis in multi-well plates using a fully recombinant, label-free reporter protein. The schematic in Figure 1A depicts the central reagent C-H-Y and its operation. In the unprocessed C-H-Y precursor, the hedgehog cholesterolysis domain (H) maintains the FRET donor, CFP, and the FRET acceptor, YFP, in proximity, resulting in a stable FRET signal. We follow the convention of reporting FRET as an emission ratio of 540 nm/460 nm after 400 nm excitation.^42^ If the “H” domain of C-H-Y is functional, the FRET signal is expected to decay in a time dependent manner following the addition of substrate (X), reporting the separation of product CFP-X from H-Y. Assays are conducted at 30 °C in 96-well plates with C-H-Y (0.2–0.1 μM) in Bis–Tris buffer (pH 7.1) and Fos-choline 12 (1.5 mM, final), the latter added as a sterol solubilizing agent. To suppress cysteine oxidation, EDTA (5 mM, final) and TCEP or DTT (4 mM, final) are included. We routinely incorporated a 10 to 30 min preincubation period with C-H-Y in complete assay buffer minus substrate to establish a starting FRET value. Following the addition of substrate, usually from an ethanol stock solution, FRET readings are recorded every 1 to 2 minutes.

#### FIGURE 1

As shown in Figure 1B, we observed stereospecific cholesterolysis activity for wild-type Xla and Dre SHhC using the FRET system. *Drosophila* Dme HhC in the C-H-Y construct was included as a control and comparator in all experiments. Stable FRET signal from samples in the preincubation period without cholesterol is consistent with successful folding of the C, H, and Y elements of the precursor protein. Cleavage of C-H-Y was apparent following the addition of excess cholesterol, 250x > [C-H-Y], which resulted in time dependent loss of FRET signal that followed pseudo-first order kinetics (Figure 1B, green symbols). The derived rate constants, k_max_, were as follows: 0.0012 sec^−1^ for *Drosophila*, and 0.0011 sec^−1^ for the zebrafish and *Xenopus* domains. Solvent control reactions where an equivalent volume of ethanol without cholesterol was added to C-H-Y samples did not appreciably alter FRET signal over the time course of the experiment (Figure 1B, black symbols). Similar inactivity was apparent in C-H-Y samples mixed with epi-cholesterol (Figure 1B, red symbols).^12^ The C1A mutants of Xla and Dre SHhC were not reactive with cholesterol or epi-cholesterol, consistent with the imperative for an N-S acyl shift to activate the G(−1) residue for cleavage (Supporting Figure 4). The D46A mutants of Xla and Dre SHhC also appeared unreactive with cholesterol up to 100 μM.

The derived rate constant for Xla SHhC cholesterolysis is in reasonable agreement with the kinetics we estimate from the study with ^35^S labeled Xla SHh precursor in egg extract. ^23^ For additional comparison, an apparent rate constant of 0.0005 sec^−1^ for Human SHhC cholesterolysis was reported from cellular pulse-chase experiments,^22^ which is within 3-fold of the values reported here.

Concentration-response plots of the initial rate of FRET loss with increasing cholesterol showed saturation behavior (Figure 1C). Derived K_M_ values of Xla SHhC and Dre SHhC were 1.3 μM and 1.1 μM, comparable to the cholesterol K_M_ value for Dme HhC (Table 1). In other enzyme systems, the K_M_ value can approximate the substrate concentration in vivo.^43^ The degree of substrate saturation for hedgehog cholesterolysis in the secretory pathway is not known. In earlier work, we observed EC50 values of ~6 μM for exogenous sterol added to chemically rescue a conditional mutant of human sonic hedgehog SHhC (D46A) expressed in HEK293 cells.^16^ A similar concentration of exogenous sterol analogs was used in mammalian cell experiments as alternative substrates for SHhC cholesterolysis.^44^ In summary, the cholesterol K_M_ values from the in vitro reconstituted SHhC assays are in the low micromolar range, in general agreement with prior cellular studies.

To validate covalent sterylation of the product “C” protein from vertebrate SHhC containing C-H-Y precursors, we used 24-azide-modified cholesterol analog (I) ^45–47^ as substrate, followed by strain-promoted click chemistry with DBCO-fluorescein (Figure 1D). Resulting protein-sterol conjugates were then analyzed by denaturing SDS-PAGE followed by in-gel fluorescence. We carried out the sterolysis and the subsequent click reaction in total soluble E. coli lysate. In-gel fluorescence of control reactions with C-H-Y that lacked sterol, or used native cholesterol, showed a non-specific pattern of fluorescein labeling (Figure 1E, *first two lanes*).^2^ By contrast, a strong, distinct fluorescent protein band was apparent in samples containing C-H-Y, the azide-sterol and DBCO-fluorescein (Figure 1E, *third lane*). The position of the fluorescent protein bands in the three gels corresponds to the “C” protein molecular weight (~30 kDa).

Taken together, results from this section indicate that recombinant SHhC domains of *Xenopus laevis* and *Danio rerio* can be expressed in *E. coli* in soluble, cholesterolysis-active form. Both vertebrate SHhC domains tolerate N- and C-terminal fusion to fluorescent proteins, enabling continuous FRET-based reporting of cholesterolysis kinetics in a multi-well format. This cleavage/sterylation activity of Xla and Dre SHhC domains with cholesterol requires their Cys1 and D46 residues, and the 3-OH group of the cholestane must have a β configuration for recognition as a substrate.

### Thioester formation by SHhC does not require cholesterol binding

#### FIGURE 2

**Figure 2.**
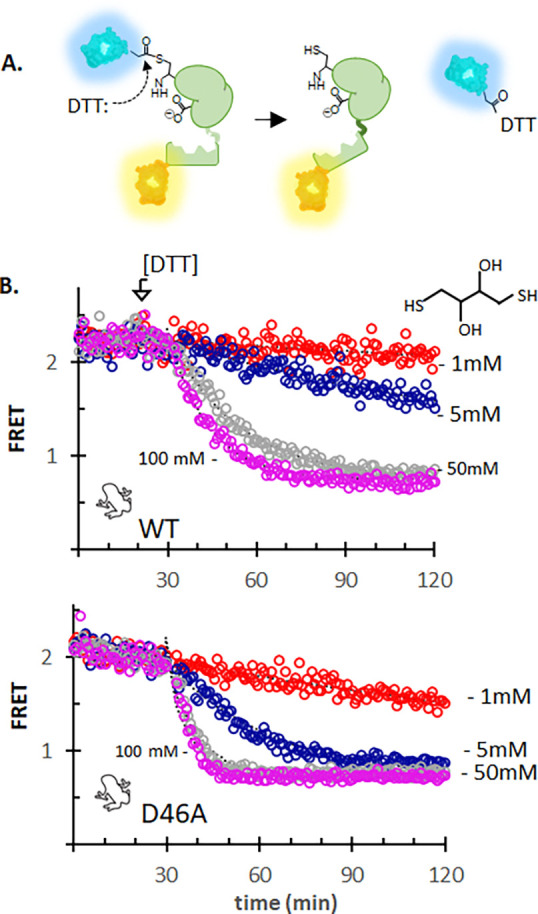
Sterol-independent thiolysis of WT and D46A SHhC precursor. **A**. Dithiothreitol (DTT) cleavage of FRET-active C-H-Y precursor into C and H-Y fragments with ensuing loss of FRET. **B**. Kinetic traces for cleavage of Xla C-H-Y wild-type (top) and Xla C-H-Y D46A (bottom) with increasing concentration of DTT. Reactions were carried out at 30 °C under identical conditions to cholesterolysis assays of Figure 1 except that cholesterol was absent.

In vitro studies of purified Drosophila Dme HhC suggested that internal thioester formation (step 1, Scheme 1) is not dependent on the presence of sterol substrate.^14^ De-acylating agents such as hydroxylamine and dithiothreitol (DTT), for example, can site-specifically cleave purified Hh precursor protein at the Gly(−1)-Cys(1) junction in sterol-free buffer (Figure 2A).^14, 48^ Although much slower than thiolysis and hydroxyaminolysis, spontaneous hydrolytic cleavage at the same Gly(−1)-Cys(1) motif has also been observed (Supporting Table 2).^17, 27^ These non-native cleavage pathways of Dme HhC are eliminated by Cys1Ala mutation. It seems reasonable to infer from these results that the Hh precursor Gly(−1)-Cys(1) bond is an equilibrium mixture of backbone amide and side chain thioester irrespective of sterol substrate.

We found that Dre SHhC and Xla SHhC precursors were likewise susceptible to DTT-induced thiolysis in the absence of cholesterol. The three wild-type C-H-Y constructs were cleaved in C and H-Y fragments by 100, 50 and 5 mM DTT in a concentration dependent manner (Supporting Figure 5). Figure 2 shows the DTT cleavage traces with Xla ShhC, WT and D46A. Zebrafish Dre SHhC reacted most rapidly with DTT, followed by Xla SHhC and Dme HhC. Dre SHhC displayed weak but measurable cleavage with 1 mM DTT, while Xla SHhC and Dme HhC were insensitive at this low DTT concentration.

Alanine substitution at D46 accelerated DTT-induced thiolysis by 2–6 fold in Dre and Xla SHhC variants. Previous structure-function studies with Dme HhC led to the proposal that the carboxylate side chain of D46 restricts N-to-S acyl shift activity at the Gly(−1)-Cys(1) motif in the absence of cholesterol. The net effect is to reduce the risk of wasteful hydrolytic cleavage of the internal Cys(1) thioester.^29^ Enhanced DTT-cleavage rates with D46A mutants of Dre and Xla SHhC suggests that this conserved residue plays a similar regulatory role for the vertebrate domains.

As expected, alanine mutation at thioester-forming Cys1 abolished DTT-induced cleavage of Dre SHhC and Xla SHhC (Supporting Figure 4). In summary, the cholesterol-independent precursor thiolysis results (Supporting Table 3) indicate that thioester formation at the Gly(−1)-Cys(1) bond and substrate binding by SHhC are not strictly coupled.

### Detergent compatibility / incompatibility for in vitro cholesterolysis

Cholesterol is sparingly soluble in aqueous solution (~1 nM), ^49^ requiring the addition of a detergent to prevent its precipitation. In the SHhC assays above, Fos-Choline 12 was used as the sterol solubilizing agent, a selection based on the results of a preliminary screen for compatible detergents we carried out using Dme HhC.

To explore the extent to which detergent structure impacts SHhC cholesterolysis activity and with a view toward future structural biology and protein biotechnology applications, we screened a commercial 96-member detergent library for SHhC compatibility. The library includes 46 non-ionic, 9 anionic, 4 cationic, and 31 zwitterionic agents, as well as six non-detergent sulfobetaines (Supporting Figure 6). Each detergent was tested in the FRET assay at their critical micelle concentration.

#### FIGURE 3

**Figure 3.**
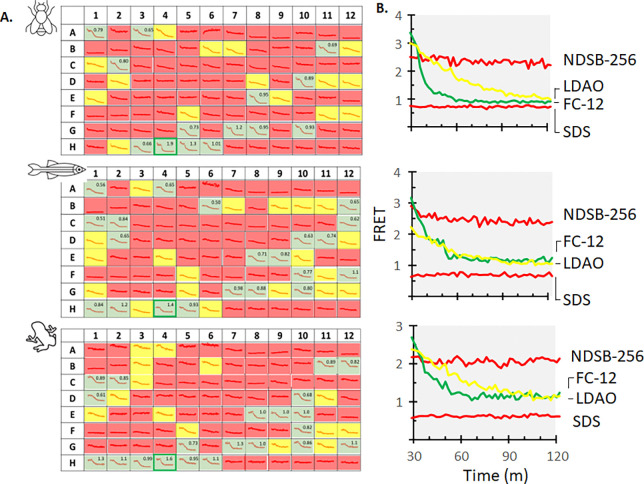
Detergent selectivity is similar for invertebrate and vertebrate cholesterolysis domains. **A**. Cholesterolysis activity for Dme, Dre and Xla domains using the C-H-Y reporter in the presence of 96 potential sterol solubilizing agents. Each detergent/surfactant was added at their critical micelle concentration. Kinetics were scored qualitatively as: fast (green); moderate (yellow); weak or no activity (red). First order rate constants (x10^−3^ sec^−1^) are shown in the upper right corner for “green” samples. The detergent fos-choline 12 supported the fastest kinetics across the three proteins (well, H4). **B**. Representative kinetic traces for green, yellow, and red detergents. Plots ordered as followed: Dme (top), Dre (middle) and Xla (bottom). Abbreviations: NDSB-256 (non-detergent sulfobetaine); LDAO (N,N-dimethyl-1-dodecanamine, N-oxide); FC-12 (n-Dodecylphosphocholine; Fos-choline-12); SDS (Sodium Dodecyl Sulfate).

Reaction progress curves for cholesterolysis were scored initially by visual inspection: *green*, rapid cholesterolysis; *yellow*, cholesterolysis active but moderate rate; *red*, no apparent cholesterol-dependent reaction (Figure 3). Some *Red* detergents, such as sodium dodecyl sulphate and sodium cholate, appeared to destabilize C-H-Y, as suggested by FRET values that started out low (<1.5) or trended lower during the assay preincubation period when cholesterol was absent. Following this qualitative assessment, kinetic data for all *green* detergents were analyzed quantitatively to obtain apparent first-order rate constant for SHhC cholesterolysis (Supporting Table 4).

Comparison of the results across the three homologous cholesterolysis domains showed similar behavior overall. Most detergents in the library were categorized as either *yellow* or *red* for weak or no activity. None of the cationic detergents were classified as *green* for high activity, and at least one (cetylpyridinium chloride, *well* **A5**) appeared inhibitory. The sulfobetaine molecules also failed to support cholesterolysis (*wells* **H7-H12**). The list of *yellow* detergents included Triton-X 100, which was used as a sterol solubilizing agent in early studies of Dme HhC cholesterolysis (*well* **E6**). Non-ionic detergents that supported more rapid cholesterolysis included nonylphenyl PEG for Dme HhC, and Tween 80 for Zebrafish and Xenopus SHhC (*wells* **E8** and **E10**). The non-ionic detergent, n-decanoyl D-sucrose, considered readily removable by dialysis, supported rapid kinetics for all three homologues (*well* **D10**). Among anionic detergents, 14:0 Lyso PG appeared best for Dme HhC, while the vertebrate Dre SHhC and Xla SHhC preferred 18:1 Lyso PG (*wells* **A1** and **A4**).

Fos-choline 12 (dodecylphosphocholine, or DPC), the synthetic zwitterionic detergent (*well* **H4**), supported the fastest kinetics for Dre SHhC, Xla SHhC and Dme HhC. In the ER, where SHhC encounters substrate cholesterol, structurally analogous phospholipids are abundant, providing a possible biological rationale for this selectivity. The effect of Foscholine 12 may involve an activating conformational change of SHhC. Preliminary concentration-response plots for C-H-Y, where FRET is plotted as a function of increasing Fos-choline 12 concentration, showed increasing FRET signal that peaks around the CMC value for Fos-choline 12 (Supporting Figure 7). A control construct, C-Y, where cyan and yellow fluorescent proteins are fused without SHhC, lacked a comparable trend in FRET signal with increasing Fos-choline 12. The molecular details of the Fos-choline 12 and SHhC interaction awaits more detailed structural analysis. To that end, Fos-choline 12 is often used for solution NMR of membrane binding proteins. Other zwitterionic detergents that scored *green* for fast kinetics with Dre SHhC, Xla SHhC and Dme HhC included 12:0 Lyso PC, 13:0 Lyso PC, and 15:0 Lyso PC, as well as Fos-choline 14 (*wells* **G7**, **G8**, **G10**, and **H5**).

To summarize, detergent structure can have a profound effect on the rate of SHhC cholesterolysis. Zwitterionic detergents appear to be the favored charge type for the invertebrate and vertebrate domains, while the cationic detergents tested here lacked SHhC compatibility. Although the overall trends are similar, we did identify species specific preferences with certain detergents (Supporting Figure 8). For in vitro assays of SHhC cholesterolysis, the zwitterionic Fos-choline 12 is recommended.

### Chemical rescue of SHhC D46A mutants with hyper-nucleophilic sterols

Chemical rescue is a mechanism-guided approach to probe enzymatic activity. A catalytically essential functional group is removed first by alanine point mutation, compromising activity toward the native substrate. Conventional chemical rescue strategies graft the missing functional group onto the substrate molecule^50^ or add the functional group in *trans* as a makeshift co-factor. ^51^ In an early example of the latter approach, a catalytic mutant of aspartate aminotransferase where alanine replaced the key general base, K258, enzymatic activity was chemically rescued by addition of exogenous aliphatic amines. ^50^ Additional examples have been reviewed.^52^ We recently introduced a third strategy for chemical rescue that uses “alpha effect” nucleophiles,^40^ which we apply again here.

#### FIGURE 4

**Figure 4.**
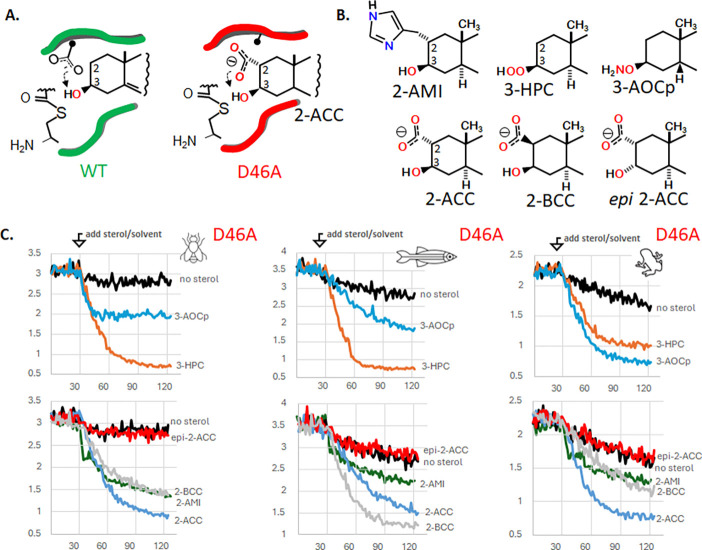
Chemical rescue of D46A mutants using hyper-nucleophilic sterols. **A**. Putative catalytic base role of conserved D46 (left); bypass pathway in D46A mutant using synthetic carboxy sterol, 2-alpha carboxy cholestanol. **B**. Chemical rescue and negative control sterols. Abbreviations: 2-AMI, 2-alpha methyl imidazole cholestanol; 3-HPC, 3-beta hydroperoxyl cholestanol; 3-AOCp, 3-beta aminoxy coprostanol; 2-ACC, 2-alpha carboxy, 3-beta hydroxy cholestanol; 2-BCC, 2-beta carboxyl, 3-beta hydroxy cholestanol; epi-2ACC, 2-alpha carboxy, 3-alpha hydroxyl cholestanol. **C**. Representative kinetic traces with D46A C-H-Y reporters for Dme, Dre and Xla domains with indicated sterol. (Top) Rescue sterols bearing alpha effect nucleophiles at the 3-position. (Bottom) Rescue sterols bearing potential general base catalysts at the 2-position. After a thirty-minute preincubation, sterols were added to a final concentration of 50 μM. Fos-choline-12 was used as the solubilizing agent.

The carboxylate group of SHhC’s D46 residue is essential for native cholesterolysis. As shown in Figure 4A, the carboxylate side chain supplies a general base to deprotonate the substrate’s 3-beta hydroxyl group. Accordingly, D46A mutants of Dme HhC and human SHhC lack measurable activity toward cholesterol. Similar inactivity was observed after D46A substitutions in Dre SHhC and Xla SHhC. As apparent from the rapid rates of precursor thiolysis by DTT (Figure 2), however, D46A variants retain their ability to form internal thioester. In two earlier studies, the first involving Dme HhC (D46A) and more recently with human SHhC(D46A) expressed in HEK293 cells, we reported that cleavage/sterylation activity could be chemically rescued by substituting cholesterol with 3-hydroperoxycholestanol (3-HPC) and 2-alpha carboxyl cholestanol(2-ACC), respectively. ^16, 40^ These nonnatural hyper-nucleophilic sterols were designed to bypass the missing general base function of D46.

Chemical rescue is extended here to Xla and Dre SHhC D46A variants using 2-ACC, 3-HPC and three new rescue sterols (Figure 4B). 2-alpha carboxyl cholestanol (2-ACC) was designed for intra-molecular general base catalysis of the sterol’s 3-β hydroxyl group. To further test this chemical rescue mechanism, we prepared 2-alpha methyl imidazole cholestanol (2-AMI) and 2-beta carboxy cholestanol (2-BCC). Similar to 2-ACC, the synthetic sterols restored activity to D46A variants of Xla and Dre SHhC as well as Dme HhC. Chemical rescue is stereospecific, as the 3-epimer of 2-ACC was inactive as a substrate. Next, we tested 3-HPC and a related analogue, 3-AOCp. The hydroperoxyl group in 3-hydroperoxycholestanol (3-HPC) is an α-effect nucleophile, where the attacking atom is adjacent (i.e., alpha) to a second electronegative atom. In a similar way, the aminoxy group of 3-aminoxycoprostanol is an α-effect nucleophile. Hydroperoxy and aminoxy groups are recognized for reacting at exceptionally fast rates compared to non-α-effect nucleophiles of equal basicity.^53–55^ We found that both 3-HPC and 3-AOCp restored activity to D46A mutants, encouraging further exploration of α-effect chemical rescue.

Conditional SHhC mutants hold promise as tools to control hedgehog cell/cell signaling in vivo. As mentioned earlier, extracellular release of the sonic hedgehog signaling ligand (SHhN) is contingent on cleavage from and sterylation by SHhC. If cholesterol and other endogenous sterols fail to serve as substrates, as with D46A mutants, the SHhN ligand is retained intracellularly and degraded. Addition of synthetic sterols that can chemically rescue D46A cleavage/sterylation activity might restore SHhN ligand release and downstream hedgehog signaling. A potential complication for this longer-term goal arises from the observed activity of 2-ACC, 2-AMI, 3-HPC, and 3-AOCp with both D46A and wild-type SHhC (Supporting Figure 9-10, Supporting table 5). In vertebrate organisms like *Xenopus laevis* and *Danio rerio* that express multiple Hh proteins, addition of non-natural sterols that promiscuously activate WT and D46A SHhC might produce broad and potentially undesired, hedgehog pathway activation. We observed a notable exception to this promiscuity with 2-BCC.

### Orthogonality of Chemical rescue with 2-beta carboxy cholestanol (2-BCC)

#### FIGURE 5

**Figure 5.**
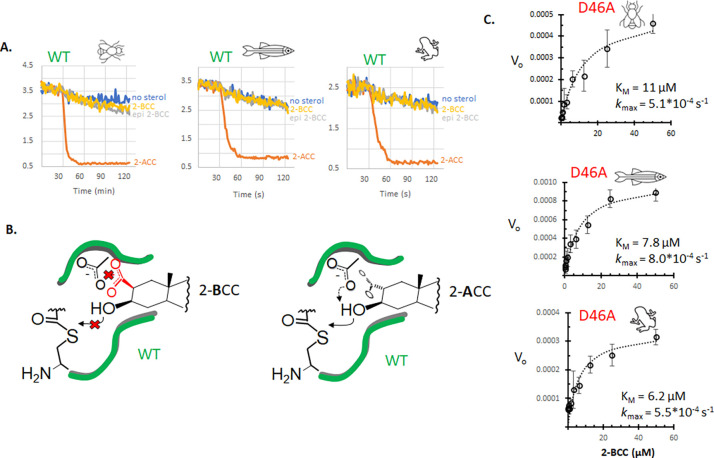
D46A-specific chemical rescue with 2-BCC. **A**. Wild-type cholesterolysis domains reject 2-BCC as a substrate. Dme, Dre and Xla C-H-Y reporter displayed robust substrate activity toward 2-ACC but not 2-BCC, or epi-2-BCC. Sterols were tested at 50 μM with Fos-choline-12 (1.5 mM, final). **B**. Proposed carboxyl-carboxyl repulsion that blocks 2-BCC but not 2-ACC from productive binding with wild-type cholesterolysis domain. **C**. Concentration response plots for 2-BCC substrate activity with invertebrate and vertebrate D46A SHhC domains along with their derived K_M_ and *k*_*max*_ values.

2-β carboxyl cholestanol showed > 100-fold selectivity for SHhC(D46A) over the wild-type protein, suggesting the possibility of truly orthogonal, mutant-specific chemical rescue. The lack of wild-type SHhC activity toward 2-BCC (Figure 5A) may arise from charge-charge repulsion between the carboxylate groups of the native D46 side chain and 2-BCC, thereby interfering with productive substrate interactions (Figure 5B). ^3^ This proposed unfavorable interaction would be absent in D46A variants. Plots of initial reaction velocity using 2-BCC as a substrate for D46A variants are shown in Figure 5C. For all three proteins, we found K_M_ and k_max_ values that were within a factor of ten of the corresponding values for cholesterol with the WT constructs. The potential for the 2-BCC/D46A pairing to manipulate sonic hedgehog ligand biogenesis and downstream signaling in complex living systems is being examined.

## CONCLUSION:

Peptide-bond cholesterolysis is a defining feature of sonic hedgehog ligand biosynthesis. While extracellular leakage of precursor hedgehog (SHhN-SHhC) has been observed in cell-culture studies, the strong genotype/phenotype association of SHhC deactivating mutations with human holoprosencephaly indicates that unprocessed hedgehog protein is inadequate for full biological signaling in vivo.^2, 3, 21, 24^ The C-terminal cholesterol molecule attached to SHhN by SHhC provides an important recognition element for extracellular SHhN transporters, assists in forming morphogenic gradients of hedgehog signaling, along with supporting functional interactions with SHhN cell surface receptors. ^56–62^

Small molecule activators of SHhC that can boost SHhN biosynthesis garner interest for mitigating hedgehog mutations linked to holoprosencephaly, whereas inhibitors of SHhC cholesterolysis activity hold promise for tamping down oncogenic SHhN biosynthesis and signaling.^16^ To date, no specific agonist or antagonist of human SHhC has been reported.

As the hedgehog founding family member, the *Drosophila melanogaster* HhC domain has long served as a valuable surrogate for understanding the molecular mechanisms of human SHhC activity. Nevertheless, sequence divergence between the human and fly proteins has motivated the pursuit of experimentally tractable SHhC domains from closer human homologues.

In this report, we established the first continuous in vitro cholesterolysis assays for SHhC domains from two vertebrate model organisms, *Danio rerio* and *Xenopus laevis*. The central reagent is a label-free, recombinant FRET-active reporter construct that monitors SHhC activity in real time, replacing more cumbersome gel-based endpoint assays. With this approach, we carried out initial kinetic characterization of vertebrate SHhC cholesterolysis and thiolysis activity, identified compatible detergents for SHhC activity, and extended the panel of unnatural sterols for chemical rescue of SHhC (D46A) mutants. Of special importance is the behavior of 2-β carboxyl cholestanol, representing the first mutantselective substrate for hedgehog precursor cleavage/sterylation.

## Supplementary Material

Supplement 1

## Figures and Tables

**Table 1.** Cholesterol substrate activity with wild-type SHhC domains at 30 °C, pH 7.1 using Fos-choline-12 (1.5 mM) as detergent.

	*k*_obs_ (s^−1^)	K_M_ (μM)
	
*Dme HhC*	1.2*10^−3^	0.69
*Dre SHhC*	1.1*10^−3^	1.1
*Xla SHhC*	1.1*10^−3^	1.3

## Footnotes

1 Recently, Wang and colleagues reported experimental evidence for an additional chemical step, preceding displacement by cholesterol, where the C(1) thioester migrates to a second conserved cysteine residue of SHhC to form a “branched intermediate”. REF 26

2 Addition of azido-PEG1 (50 μM, final) to control reactions lacking the sterol-azide probe (lanes 1, 2), eliminated this unspecific fluorescence labeling.

3 Consistent with anion-anion charge repulsion, initial characterization of an analog of 2-BCC where the carboxylate group is replaced with an imidazole group, does not show this same mutant selectivity; the 2-β imidazolyl analog is accepted by both D46A and WT proteins, with enhanced reactivity toward WT (Supporting Figure 10).

## References

[1] Zhang Y.; Beachy P. A., Cellular and molecular mechanisms of Hedgehog signalling. Nat Rev Mol Cell Biol 2023, 24 (9), 668–687. 36932157 10.1038/s41580-023-00591-1 PMC12140928

[2] Belloni E.; Muenke M.; Roessler E.; Traverso G.; Siegel-Bartelt J.; Frumkin A.; Mitchell H. F.; Donis-Keller H.; Helms C.; Hing A. V.; Heng H. H.; Koop B.; Martindale D.; Rommens J. M.; Tsui L. C.; Scherer S. W., Identification of Sonic hedgehog as a candidate gene responsible for holoprosencephaly. Nat Genet 1996, 14 (3), 353–6. 8896571 10.1038/ng1196-353

[3] Hong S.; Hu P.; Jang J. H.; Carrington B.; Sood R.; Berger S. I.; Roessler E.; Muenke M., Functional analysis of Sonic Hedgehog variants associated with holoprosencephaly in humans using a CRISPR/Cas9 zebrafish model. Hum Mutat 2020, 41 (12), 2155–2166. 32939873 10.1002/humu.24119

[4] Wang X.; Wang T.; Song X.; Gao J.; Xu G.; Ma Y.; Song G., Current Status of Hedgehog Signaling Inhibitors. Curr Top Med Chem 2024, 24 (3), 243–258. 38231069 10.2174/0115680266280850231221074340

[5] Vaghjiani V. G.; Cochrane C. R.; Jayasekara W. S. N.; Chong W. C.; Szczepny A.; Kumar B.; Martelotto L. G.; McCaw A.; Carey K.; Kansara M.; Thomas D. M.; Walkley C.; Mudge S.; Gough D. J.; Downie P. A.; Peacock C. D.; Matsui W.; Watkins D. N.; Cain J. E., Ligand-dependent hedgehog signaling maintains an undifferentiated, malignant osteosarcoma phenotype. Oncogene 2023, 42 (47), 3529–3541. 37845394 10.1038/s41388-023-02864-7 PMC10656285

[6] Jing J.; Wu Z.; Wang J.; Luo G.; Lin H.; Fan Y.; Zhou C., Hedgehog signaling in tissue homeostasis, cancers, and targeted therapies. Signal Transduct Target Ther 2023, 8 (1), 315. 37596267 10.1038/s41392-023-01559-5 PMC10439210

[7] Lee J. J.; Ekker S. C.; von Kessler D. P.; Porter J. A.; Sun B. I.; Beachy P. A., Autoproteolysis in hedgehog protein biogenesis. Science 1994, 266 (5190), 1528–37. 7985023 10.1126/science.7985023

[8] Perler F. B., Protein splicing of inteins and hedgehog autoproteolysis: structure, function, and evolution. Cell 1998, 92 (1), 1–4. 10.1016/s0092-8674(00)80892-2 9489693

[9] Marti E.; Bumcrot D. A.; Takada R.; McMahon A. P., Requirement of 19K form of Sonic hedgehog for induction of distinct ventral cell types in CNS explants. Nature 1995, 375 (6529), 322–5. 7753196 10.1038/375322a0

[10] Bumcrot D. A.; Takada R.; McMahon A. P., Proteolytic processing yields two secreted forms of sonic hedgehog. Mol Cell Biol 1995, 15 (4), 2294–303. 7891723 10.1128/mcb.15.4.2294 PMC230457

[11] Burglin T. R., Warthog and groundhog, novel families related to hedgehog. Curr Biol 1996, 6 (9), 1047–50. 10.1016/s0960-9822(02)70659-3 8805384

[12] Mann R. K.; Beachy P. A., Cholesterol modification of proteins. Biochim Biophys Acta 2000, 1529 (1–3), 188–202. 11111088 10.1016/s1388-1981(00)00148-7

[13] Ciepla P.; Magee A. I.; Tate E. W., Cholesterylation: a tail of hedgehog. Biochem Soc Trans 2015, 43 (2), 262–7. 25849927 10.1042/BST20150032

[14] Hall T. M.; Porter J. A.; Young K. E.; Koonin E. V.; Beachy P. A.; Leahy D. J., Crystal structure of a Hedgehog autoprocessing domain: homology between Hedgehog and self-splicing proteins. Cell 1997, 91 (1), 85–97. 9335337 10.1016/s0092-8674(01)80011-8

[15] Porter J. A.; Young K. E.; Beachy P. A., Cholesterol modification of hedgehog signaling proteins in animal development. Science 1996, 274 (5285), 255–9. 8824192 10.1126/science.274.5285.255

[16] Ciulla D. A.; Dranchak P.; Pezzullo J. L.; Mancusi R. A.; Psaras A. M.; Rai G.; Giner J. L.; Inglese J.; Callahan B. P., A cell-based bioluminescence reporter assay of human Sonic Hedgehog protein autoprocessing to identify inhibitors and activators. J Biol Chem 2022, 298 (12), 102705. 10.1016/j.jbc.2022.102705 PMC9772569 36400200

[17] Owen T. S.; Ngoje G.; Lageman T. J.; Bordeau B. M.; Belfort M.; Callahan B. P., Forster resonance energy transfer-based cholesterolysis assay identifies a novel hedgehog inhibitor. Anal Biochem 2015, 488, 1–5. 26095399 10.1016/j.ab.2015.06.021 PMC4591182

[18] Zhang X.; Kotikam V.; Rozners E.; Callahan B. P., Enzymatic Beacons for Specific Sensing of Dilute Nucleic Acid. Chembiochem 2022, 23 (4), e202100594. 10.1002/cbic.202100594 PMC8961972 34890095

[19] Mozhdehi D.; Luginbuhl K. M.; Dzuricky M.; Costa S. A.; Xiong S.; Huang F. C.; Lewis M. M.; Zelenetz S. R.; Colby C. D.; Chilkoti A., Genetically Encoded Cholesterol-Modified Polypeptides. J Am Chem Soc 2019, 141 (2), 945–951. 30608674 10.1021/jacs.8b10687 PMC6693588

[20] Hossain M. S.; Liu X.; Maynard T. I.; Mozhdehi D., Genetically Encoded Inverse Bolaamphiphiles. Biomacromolecules 2020, 21 (2), 660–669. 31855430 10.1021/acs.biomac.9b01380

[21] Traiffort E.; Dubourg C.; Faure H.; Rognan D.; Odent S.; Durou M. R.; David V.; Ruat M., Functional characterization of sonic hedgehog mutations associated with holoprosencephaly. J Biol Chem 2004, 279 (41), 42889–97. 15292211 10.1074/jbc.M405161200

[22] Guy R. K., Inhibition of sonic hedgehog autoprocessing in cultured mammalian cells by sterol deprivation. Proc Natl Acad Sci U S A 2000, 97 (13), 7307–12. 10860995 10.1073/pnas.97.13.7307 PMC16541

[23] Chen X.; Tukachinsky H.; Huang C. H.; Jao C.; Chu Y. R.; Tang H. Y.; Mueller B.; Schulman S.; Rapoport T. A.; Salic A., Processing and turnover of the Hedgehog protein in the endoplasmic reticulum. J Cell Biol 2011, 192 (5), 825–38. 21357747 10.1083/jcb.201008090 PMC3051819

[24] Maity T.; Fuse N.; Beachy P. A., Molecular mechanisms of Sonic hedgehog mutant effects in holoprosencephaly. Proc Natl Acad Sci U S A 2005, 102 (47), 17026–31. 16282375 10.1073/pnas.0507848102 PMC1282174

[25] Bordeau B. M.; Ciulla D. A.; Callahan B. P., Hedgehog Proteins Consume Steroidal CYP17A1 Antagonists: Potential Therapeutic Significance in Advanced Prostate Cancer. ChemMedChem 2016, 11 (18), 1983–6. 27435344 10.1002/cmdc.201600238 PMC5588864

[26] Faris S.; Xia K.; Wagner A. G.; Xu Z.; Smith N.; Giner J. L.; Callahan B.; Xie J.; Wang C., Conserved C143 forms a branched intermediate in Hedgehog autoprocessing: A cancer drug discovery target against Hedgehog signaling. Proc Natl Acad Sci U S A 2025, 122 (17), e2415144122. 10.1073/pnas.2415144122 PMC12054796 40273103

[27] Smith C. J.; Wagner A. G.; Stagnitta R. T.; Xu Z.; Pezzullo J. L.; Giner J. L.; Xie J.; Covey D. F.; Wang C.; Callahan B. P., Subverting Hedgehog Protein Autoprocessing by Chemical Induction of Paracatalysis. Biochemistry 2020, 59 (6), 736–741. 32013401 10.1021/acs.biochem.0c00013 PMC7031038

[28] Wagner A. G.; Stagnitta R. T.; Xu Z.; Pezzullo J. L.; Kandel N.; Giner J. L.; Covey D. F.; Wang C.; Callahan B. P., Nanomolar, Noncovalent Antagonism of Hedgehog Cholesterolysis: Exception to the “Irreversibility Rule” for Protein Autoprocessing Inhibition. Biochemistry 2022, 61 (11), 1022–1028. 34941260 10.1021/acs.biochem.1c00697 PMC9382716

[29] Xie J.; Owen T.; Xia K.; Callahan B.; Wang C., A Single Aspartate Coordinates Two Catalytic Steps in Hedgehog Autoprocessing. J Am Chem Soc 2016, 138 (34), 10806–9. 27529645 10.1021/jacs.6b06928 PMC5589136

[30] Xie J.; Du Z.; Callahan B.; Belfort M.; Wang C., (1)H, (1)(3)C and (1)(5)N NMR assignments of a Drosophila Hedgehog autoprocessing domain. Biomol NMR Assign 2014, 8 (2), 279–81. 23765287 10.1007/s12104-013-9500-8 PMC3884045

[31] Zhao J.; Ciulla D. A.; Xie J.; Wagner A. G.; Castillo D. A.; Zwarycz A. S.; Lin Z.; Beadle S.; Giner J. L.; Li Z.; Li H.; Banavali N.; Callahan B. P.; Wang C., General Base Swap Preserves Activity and Expands Substrate Tolerance in Hedgehog Autoprocessing. J Am Chem Soc 2019, 141 (46), 18380–18384. 31682419 10.1021/jacs.9b08914 PMC7106946

[32] Huang C. H.; Hsiao H. T.; Chu Y. R.; Ye Y.; Chen X., Derlin2 protein facilitates HRD1-mediated retro-translocation of sonic hedgehog at the endoplasmic reticulum. J Biol Chem 2013, 288 (35), 25330–9. 23867461 10.1074/jbc.M113.455212 PMC3757197

[33] Clark A. J.; Block K., The absence of sterol synthesis in insects. J Biol Chem 1959, 234, 2578–82. 13810427

[34] Stenkamp D. L.; Frey R. A.; Prabhudesai S. N.; Raymond P. A., Function for Hedgehog genes in zebrafish retinal development. Dev Biol 2000, 220 (2), 238–52. 10753513 10.1006/dbio.2000.9629

[35] Jackman W. R.; Yoo J. J.; Stock D. W., Hedgehog signaling is required at multiple stages of zebrafish tooth development. BMC Dev Biol 2010, 10, 119. 21118524 10.1186/1471-213X-10-119 PMC3001715

[36] Male I.; Ozacar A. T.; Fagan R. R.; Loring M. D.; Shen M. C.; Pace V. A.; Devine C. A.; Lawson G. E.; Lutservitz A.; Karlstrom R. O., Hedgehog Signaling Regulates Neurogenesis in the Larval and Adult Zebrafish Hypothalamus. eNeuro 2020, 7 (6). 10.1523/ENEURO.0226-20.2020 PMC7769882 33106384

[37] Yin A.; Winata C. L.; Korzh S.; Korzh V.; Gong Z., Expression of components of Wnt and Hedgehog pathways in different tissue layers during lung development in Xenopus laevis. Gene Expr Patterns 2010, 10 (7–8), 338–44. 20682360 10.1016/j.gep.2010.07.005

[38] Dominguez L.; Gonzalez A.; Moreno N., Sonic hedgehog expression during Xenopus laevis forebrain development. Brain Res 2010, 1347, 19–32. 20540934 10.1016/j.brainres.2010.06.007

[39] Hamilton A. M.; Balashova O. A.; Borodinsky L. N., Non-canonical Hedgehog signaling regulates spinal cord and muscle regeneration in Xenopus laevis larvae. Elife 2021, 10. 10.7554/eLife.61804 PMC8137141 33955353

[40] Ciulla D. A.; Jorgensen M. T.; Giner J. L.; Callahan B. P., Chemical Bypass of General Base Catalysis in Hedgehog Protein Cholesterolysis Using a Hyper-Nucleophilic Substrate. J Am Chem Soc 2018, 140 (3), 916–918. 28930454 10.1021/jacs.7b05161 PMC6054137

[41] Ciepla P.; Konitsiotis A. D.; Serwa R. A.; Masumoto N.; Leong W. P.; Dallman M. J.; Magee A. I.; Tate E. W., New chemical probes targeting cholesterylation of Sonic Hedgehog in human cells and zebrafish. Chem Sci 2014, 5 (11), 4249–4259. 25574372 10.1039/c4sc01600a PMC4285107

[42] Amitai G.; Callahan B. P.; Stanger M. J.; Belfort G.; Belfort M., Modulation of intein activity by its neighboring extein substrates. Proc Natl Acad Sci U S A 2009, 106 (27), 11005–10. 19541659 10.1073/pnas.0904366106 PMC2708771

[43] Bennett B. D.; Kimball E. H.; Gao M.; Osterhout R.; Van Dien S. J.; Rabinowitz J. D., Absolute metabolite concentrations and implied enzyme active site occupancy in Escherichia coli. Nat Chem Biol 2009, 5 (8), 593–9. 19561621 10.1038/nchembio.186 PMC2754216

[44] Heal W. P.; Jovanovic B.; Bessin S.; Wright M. H.; Magee A. I.; Tate E. W., Bioorthogonal chemical tagging of protein cholesterylation in living cells. Chem Commun (Camb) 2011, 47 (14), 4081–3. 21221452 10.1039/c0cc04710d

[45] Xiao X.; Tang J. J.; Peng C.; Wang Y.; Fu L.; Qiu Z. P.; Xiong Y.; Yang L. F.; Cui H. W.; He X. L.; Yin L.; Qi W.; Wong C. C.; Zhao Y.; Li B. L.; Qiu W. W.; Song B. L., Cholesterol Modification of Smoothened Is Required for Hedgehog Signaling. Mol Cell 2017, 66 (1), 154–162 e10. 28344083 10.1016/j.molcel.2017.02.015

[46] Lorizate M.; Terrones O.; Nieto-Garai J. A.; Rojo-Bartolome I.; Ciceri D.; Morana O.; Olazar-Intxausti J.; Arboleya A.; Martin A.; Szynkiewicz M.; Calleja-Felipe M.; Bernardino de la Serna J.; Contreras F. X., Super-Resolution Microscopy Using a Bioorthogonal-Based Cholesterol Probe Provides Unprecedented Capabilities for Imaging Nanoscale Lipid Heterogeneity in Living Cells. Small Methods 2021, 5 (9), e2100430. 10.1002/smtd.202100430 34928061

[47] Hu A.; Zhou M.; Song B. L., Analysis of Protein Cholesterylation by Biorthogonal Labeling. *Methods* Mol Biol 2022, 2374, 27–36. 10.1007/978-1-0716-1701-4_3 34562240

[48] Xie J.; Owen T.; Xia K.; Singh A. V.; Tou E.; Li L.; Arduini B.; Li H.; Wan L. Q.; Callahan B.; Wang C., Zinc inhibits Hedgehog autoprocessing: linking zinc deficiency with Hedgehog activation. J Biol Chem 2015, 290 (18), 11591–600. 25787080 10.1074/jbc.M114.623264 PMC4416862

[49] Haberland M. E.; Reynolds J. A., Self-association of cholesterol in aqueous solution. Proc Natl Acad Sci U S A 1973, 70 (8), 2313–6. 4525165 10.1073/pnas.70.8.2313 PMC433725

[50] Toney M. D.; Kirsch J. F., Direct Bronsted analysis of the restoration of activity to a mutant enzyme by exogenous amines. Science 1989, 243 (4897), 1485–8. 2538921 10.1126/science.2538921

[51] Carter P.; Wells J. A., Engineering enzyme specificity by “substrate-assisted catalysis”. Science 1987, 237 (4813), 394–9. 3299704 10.1126/science.3299704

[52] O’Meara T. R.; Palanski B. A.; Chen M.; Qiao Y.; Cole P. A., Mutant protein chemical rescue: From mechanisms to therapeutics. J Biol Chem 2025, 301 (4), 108417. 10.1016/j.jbc.2025.108417 PMC12018205 40113044

[53] Dixon J. E.; Bruice T. C., Alpha-Effect .5. Kinetic and Thermodynamic Nature of Alpha-Effect for Amine Nucleophiles. Journal of the American Chemical Society 1972, 94 (6), 2052-&.

[54] Herschlag D.; Jencks W. P., Nucleophiles of High Reactivity in Phosphoryl Transfer-Reactions - Alpha-Effect Compounds and Fluoride-Ion. Journal of the American Chemical Society 1990, 112 (5), 1951–1956.

[55] Inward P. W.; Jencks W. P., Reactivity of Nucleophilic Reagents with Furoyl-Chymotrypsin. Journal of Biological Chemistry 1965, 240 (5), 1986-+. 14299619

[56] Creanga A.; Glenn T. D.; Mann R. K.; Saunders A. M.; Talbot W. S.; Beachy P. A., Scube/You activity mediates release of dually lipid-modified Hedgehog signal in soluble form. Genes Dev 2012, 26 (12), 1312–25. 22677548 10.1101/gad.191866.112 PMC3387659

[57] Tukachinsky H.; Kuzmickas R. P.; Jao C. Y.; Liu J.; Salic A., Dispatched and scube mediate the efficient secretion of the cholesterol-modified hedgehog ligand. Cell Rep 2012, 2 (2), 308–20. 22902404 10.1016/j.celrep.2012.07.010 PMC3682496

[58] Burke R.; Nellen D.; Bellotto M.; Hafen E.; Senti K. A.; Dickson B. J.; Basler K., Dispatched, a novel sterol-sensing domain protein dedicated to the release of cholesterol-modified hedgehog from signaling cells. Cell 1999, 99 (7), 803–15. 10619433 10.1016/s0092-8674(00)81677-3

[59] Rudolf A. F.; Kinnebrew M.; Kowatsch C.; Ansell T. B.; El Omari K.; Bishop B.; Pardon E.; Schwab R. A.; Malinauskas T.; Qian M.; Duman R.; Covey D. F.; Steyaert J.; Wagner A.; Sansom M. S. P.; Rohatgi R.; Siebold C., The morphogen Sonic hedgehog inhibits its receptor Patched by a pincer grasp mechanism. Nat Chem Biol 2019, 15 (10), 975–982. 31548691 10.1038/s41589-019-0370-y PMC6764859

[60] Pepinsky R. B.; Zeng C.; Wen D.; Rayhorn P.; Baker D. P.; Williams K. P.; Bixler S. A.; Ambrose C. M.; Garber E. A.; Miatkowski K.; Taylor F. R.; Wang E. A.; Galdes A., Identification of a palmitic acid-modified form of human Sonic hedgehog. J Biol Chem 1998, 273 (22), 14037–45. 9593755 10.1074/jbc.273.22.14037

[61] Grover V. K.; Valadez J. G.; Bowman A. B.; Cooper M. K., Lipid modifications of Sonic hedgehog ligand dictate cellular reception and signal response. PLoS One 2011, 6 (7), e21353. 10.1371/journal.pone.0021353 PMC3128587 21747935

[62] Schlissel G.; Meziane M.; Narducci D.; Hansen A. S.; Li P., Diffusion barriers imposed by tissue topology shape Hedgehog morphogen gradients. Proc Natl Acad Sci U S A 2024, 121 (36), e2400677121. 10.1073/pnas.2400677121 PMC11388384 39190357

