# Supplementary material for: In vitro reconstitution of vertebrate Sonic Hedgehog protein cholesterolysis
Source: bioRxiv. 2026 Mar 11:2026.03.09.710561. Preprint. [Version 1] doi: 10.64898/2026.03.09.710561 (PMC13060885; doi:10.64898/2026.03.09.710561)
Supplement: Supplement 1 [file NIHPP2026.03.09.710561v1-supplement-1.pdf]

bioRxiv preprint doi: https://doi.org/10.64898/2026.03.09.710561; this version posted March 11, 2026. The copyright holder for this preprint (which was not certified by peer review) is the author/funder, who has granted bioRxiv a license to display the preprint in perpetuity. It is made available under aCC-BY 4.0 International license.

Supporting Information

In vitro reconstitution of vertebrate Sonic Hedgehog protein cholesterolysis

Dayton C. Seidel[1‡], Andrew G. Wagner[1‡], John L. Pezzullo[2], Katherine A. Thayer[2], Seth Beadle[2], Margot L. Olejarczyk[2], José-Luis Giner[2*], Brian P. Callahan[1*]

[1]Department of Chemistry, Binghamton University, State University of New York, Binghamton, New York 13902, United States

[2]Department of Chemistry, State University of New York College of Environmental Science and Forestry, Syracuse, New York 13210, United States

[‡] Equal contribution

*Corresponding Authors: Brian P. Callahan — Department of Chemistry, Binghamton University, State University of New York, Binghamton, New York 13902, United States; callahan@binghamton.edu; José-Luis Giner — Department of Chemistry, State University of New York College of Environmental Science and Forestry, Syracuse, New York 13210, United States; jlginer@syr.edu

Supporting Tables S2

Supporting Figures S7

Supporting Methods S17

1. Protein preparation and assay methods S18
   - Construction of FRET reporter constructs for Xla and Dru SHhC S18
   - *E. coli* expression and NI-NTA purification of C-H-Y FRET reporter S18
   - FRET assays for SHhC cholesterolysis S18-19
   - Determination of k_{max} and K_M values from FRET assay data S19
   - Chemical Rescue S19
   - Detergent screening for compatibility with SHhC cholesterolysis S19
   - Thiolysis of SHhC precursor using the FRET reporter S19

2. Chemical synthesis of the rescue sterols S20
   - General procedures S20
   - Synthesis of 2-carboxycholestanol isomers S20-22
   - Synthesis of 2-imidazolemethylcholestanol isomers S22-25
   - Synthesis of 3β-aminoxy-5β-cholestane (2-BMI) S25-26
   - Synthesis of 24-azido-5α-chol-5-en-3β-ol S26-28

bioRxiv preprint doi: https://doi.org/10.64898/2026.03.09.710561; this version posted March 11, 2026. The copyright holder for this preprint (which was not certified by peer review) is the author/funder, who has granted bioRxiv a license to display the preprint in perpetuity. It is made available under aCC-BY 4.0 International license.

Supporting Tables

Table S1. Sequence identity and similarity of the cholesterolysis domains from selected model organisms compared to human SHhC[a]

	Dme HhC	Dre SHhC	Xla SHhC
Identity	32%	46%	49%
Similarity	45%	60%	59%

[a] Identity and similarity were found by Blast2 sequence alignment using SHhC sequences from UniProt, and cited in **supporting figure 1**

Table S2. Apparent rates of spontaneous precursor hydrolysis at 30 °C, pH 7.1, for *Drosophila* (Dme), *Danio* (Dre), and *Xenopus* (Xla) Wild-Type and D46A HhC using the FRET reporter C-H-Y

Construct	k_{max} (s^{-1})	$t_{1/2}$ (hrs)
Dme HhC (WT)	$0.52*10^{-5}$	37
Dre HhC (WT)	$5.0*10^{-5}$	3.9
Xla HhC (WT)	$3.2*10^{-5}$	6.0
Dme HhC (D46A)	$0.65*10^{-5}$	30
Dre HhC (D46A)	$5.4*10^{-5}$	3.6
Xla HhC (D46A)	$4.4*10^{-5}$	4.4

bioRxiv preprint doi: https://doi.org/10.64898/2026.03.09.710561; this version posted March 11, 2026. The copyright holder for this preprint (which was not certified by peer review) is the author/funder, who has granted bioRxiv a license to display the preprint in perpetuity. It is made available under aCC-BY 4.0 International license.

Table S3. Thiolysis activity with Dithiothreitol (DTT) for Dme, Dre, and Xla WT and D46A SHhC using FRET reporter

	k_{max} (s^{-1})			
Construct	100mM DTT	50mM DTT	5mM DTT	1mM DTT
Dme HhC (WT)	$6.0*10^{-4}$	$3.4*10^{-4}$	$0.53*10^{-4}$	0.16×10^{-4}
Dre HhC (WT)	$21*10^{-4}$	$14*10^{-4}$	$3.5*10^{-4}$	$0.93*10^{-4}$
Xla HhC (WT)	$12*10^{-4}$	$6.9*10^{-4}$	$1.2*10^{-4}$	$<0.32 \times 10^{-4}$
Dme HhC (D46A)	$14*10^{-4}$	$9.0*10^{-4}$	$1.4*10^{-4}$	0.30×10^{-4}
Dre HhC (D46A)	$28*10^{-4}$	$22*10^{-4}$	$11*10^{-4}$	$2.9*10^{-4}$
Xla HhC (D46A)	$23*10^{-4}$	$16*10^{-4}$	$5.5*10^{-4}$	$0.85*10^{-4}$

bioRxiv preprint doi: https://doi.org/10.64898/2026.03.09.710561; this version posted March 11, 2026. The copyright holder for this preprint (which was not certified by peer review) is the author/funder, who has granted bioRxiv a license to display the preprint in perpetuity. It is made available under aCC-BY 4.0 International license.

Table S4. Compatible detergents for cholesterolysis with Dme, Dre, and Xla SHhC at 30°C, in Bis-Tris buffer (pH 7.1)

Drosophila			*Danio*			*Xenopus*		
Detergent[a]	Type	k_{obs} (s^{-1})	Detergent[a]	Type	k_{obs} (s^{-1})	Detergent[a]	Type	k_{obs} (s^{-1})
14:0 Lyso PG	I	0.79*10^{-3}	14:0 Lyso PG	I	0.56*10^{-3}	Brij 35	N	0.89*10^{-3}
18:0 Lyso PG	I	0.65*10^{-3}	18:1 Lyso PG	I	0.65*10^{-3}	Brij 56	N	0.82*10^{-3}
Brij 35	N	0.69*10^{-3}	APO 12	N	0.50*10^{-3}	Brij 58	N	0.89*10^{-3}
Genapol X-080	N	0.80*10^{-3}	Brij 56	N	0.65*10^{-3}	Genapol X-080	N	0.85*10^{-3}
n-Decanoylsucrose	N	0.89*10^{-3}	Brij 58	N	0.51*10^{-3}	DDAO	N	0.61*10^{-3}
Nonylphenyl polyethylene glycol	N	0.95*10^{-3}	Genapol X-080	N	0.84*10^{-3}	n-Decanoylsucrose	N	0.68*10^{-3}
10:0 Lyso PC	Z	0.73*10^{-3}	Mal(11.2)	N	0.62*10^{-3}	Nonylphenyl polyethylene glycol	N	1.0*10^{-3}
12:0 Lyso PC	Z	1.2*10^{-3}	n-Decyl-β-D-maltoside	N	0.65*10^{-3}	Tween 20	N	1.0*10^{-3}
13:0 Lyso PC	Z	0.95*10^{-3}	n-Decanoylsucrose	N	0.63*10^{-3}	Tween 80	N	1.0*10^{-3}
15:0 Lyso PC	Z	0.93*10^{-3}	N-Nonyl-β-D-glucoside	N	0.74*10^{-3}	Zwittergent 3-16	N	0.82*10^{-3}
Fos-Choline 10	Z	0.66*10^{-3}	Nonylphenyl polyethylene glycol	N	0.71*10^{-3}	10:0 Lyso PC	Z	0.73*10^{-3}
Fos-Choline 12	Z	1.9*10^{-3}	Tween 20	N	0.82*10^{-3}	12:0 Lyso PC	Z	1.3*10^{-3}
Fos-Choline 14	Z	1.3*10^{-3}	Zwittergent 3-16	N	1.0*10^{-3}	13:0 Lyso PC	Z	1.0*10^{-3}
Fos-Choline 16	Z	1.0*10^{-3}	07:0 PC (DHPC)	Z	1.1*10^{-3}	15:0 Lyso PC	Z	0.86*10^{-3}

[a] Detergents plate designations can be found in **supporting figure 6**

bioRxiv preprint doi: https://doi.org/10.64898/2026.03.09.710561; this version posted March 11, 2026. The copyright holder for this preprint (which was not certified by peer review) is the author/funder, who has granted bioRxiv a license to display the preprint in perpetuity. It is made available under aCC-BY 4.0 International license.

Table S4 Continued.

Danio			*Xenopus*		
Detergent[a]	Type	k_{obs} (s^{-1})	Detergent[a]	Type	k_{obs} (s^{-1})
12:0 Lyso PC	Z	$0.98*10^{-3}$	17:0 Lyso PC	Z	$1.1*10^{-3}$
13:0 Lyso PC	Z	$0.88*10^{-3}$	18:0 Lyso PC	Z	$1.3*10^{-3}$
15:0 Lyso PC	Z	$0.80*10^{-3}$	18:1 Lyso PC	Z	$1.1*10^{-3}$
18:0 Lyso PC	Z	$0.84*10^{-3}$	Fos-Choline 10	Z	$0.99*10^{-3}$
18:1 Lyso PC	Z	$1.2*10^{-3}$	Fos-Choline 12	Z	$1.6*10^{-3}$
Fos-Choline 12	Z	$1.4*10^{-3}$	Fos-Choline 14	Z	$0.95*10^{-3}$
Fos-Choline 14	Z	$0.93*10^{-3}$	Fos-Choline 16	Z	$1.1*10^{-3}$

[a] Detergents plate designations can be found in **supporting figure 6**

bioRxiv preprint doi: https://doi.org/10.64898/2026.03.09.710561; this version posted March 11, 2026. The copyright holder for this preprint (which was not certified by peer review) is the author/funder, who has granted bioRxiv a license to display the preprint in perpetuity. It is made available under aCC-BY 4.0 International license.

Table S5. Substrate activity of engineered sterols with *Drosophila*, *Danio*, and *Xenopus* Wild-Type HhC

		k_{max} (s^{-1})		
Substrate	Structure	Dme HhC	Dre SHhC	Xla SHhC
3-AOCp	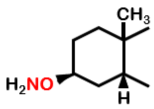	0.86×10^{-3}	$<5.0*10^{-5}$	0.14×10^{-3}
3-HPC	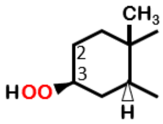	1.3×10^{-3}	0.64×10^{-3}	0.62×10^{-3}
2-AMI	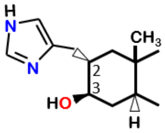	0.82×10^{-3}	0.19×10^{-3}	0.49×10^{-3}
2-ACC	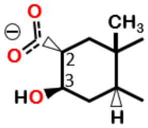	3.8×10^{-3}	2.3×10^{-3}	1.7×10^{-3}
epi 2-ACC	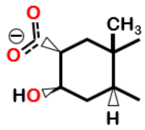	$\leq 4.6*10^{-5}$	$\leq 5.2*10^{-5}$	$\leq 4.0*10^{-5}$
2-BCC	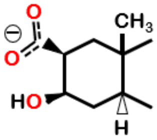	$\leq 3.4*10^{-5}$	$\leq 5.0*10^{-5}$	$\leq 3.8*10^{-5}$

bioRxiv preprint doi: https://doi.org/10.64898/2026.03.09.710561; this version posted March 11, 2026. The copyright holder for this preprint (which was not certified by peer review) is the author/funder, who has granted bioRxiv a license to display the preprint in perpetuity. It is made available under aCC-BY 4.0 International license.

Supporting Figures

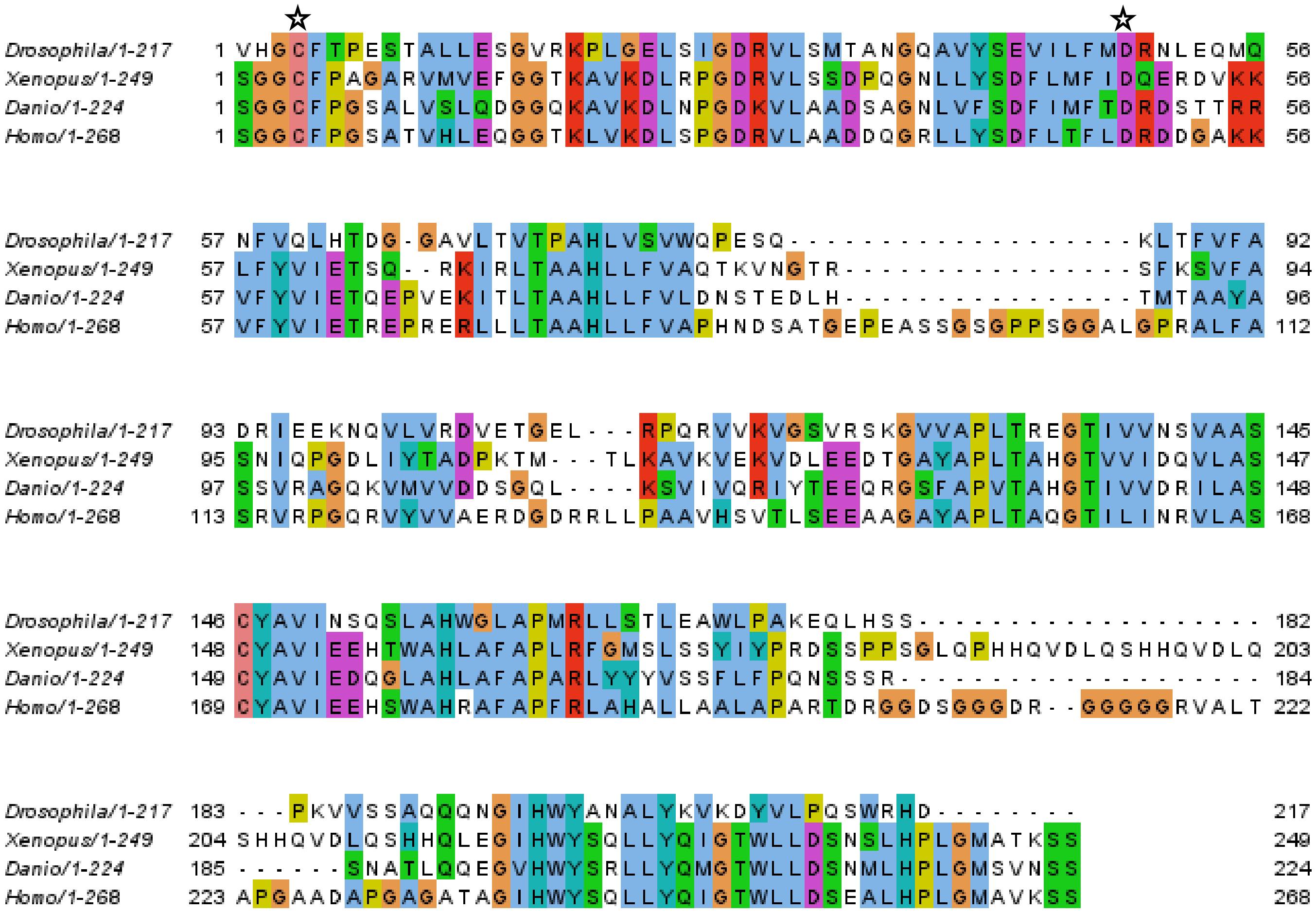

Figure S1. Sequence alignment of *Drosophila*, *Xenopus*, *Danio*, and human SHhC. Uniprot accession numbers: Q02936 (*Drosophila*), Q92008 (*Danio*), Q92000 (*Xenopus*), Q15465 (*Homo*). The identity and similarity values are summarized in **supporting table 1**. The catalytically essential C1 and D46 residues are indicated with a star. Note that the (-3) residue here is numbered as (1).

bioRxiv preprint doi: https://doi.org/10.64898/2026.03.09.710561; this version posted March 11, 2026. The copyright holder for this preprint (which was not certified by peer review) is the author/funder, who has granted bioRxiv a license to display the preprint in perpetuity. It is made available under aCC-BY 4.0 International license.

Amino acid sequence of *Drosophila* C-H-Y:

MSKGEELFGGIVPILVELEGDVNGHKFSVSGEGEGDATYGKLTLKFICTTGKLPVPWPTLVTTLTWGVQCFSRYPDHMKQHDFFKSV
MPEGYVQERTIFFKDDGNYKTRAEVKFEGDTLVNRIELKGIDFKEDGNILGHKLEYNYISHNVYITADKQKNGIKANFKARHNITDGS
VQLADHYQQNTPIGDGPVILPDNHYLSTQSALSKDPNEKRDHMVLLEFVTAAGITHGMDELYKGSGGSTVHG**C**FTPESTALLESGV
RKPLGELSIGDRVLSMTANGQAVYSEVILFM**D**RNLEQMQNFVQLHTDGGAVLTVTPAHLVSVWQPESQKLTFVFADRIEEKNQVL
VRDVETGELRPQRVVKVGSVRSKGVVAPLTREGTIVVNSVAASCYAVINSQSLAHWGLAPMRLLSTLEAWLPAKEQLHSSPKVVSSA
QQQNGIHWYANALYKVKDYVLPQSWRHDGGLQGSGGSMSKGEELFTGVVPILVELDGDVNGHKFSVSGEGEGDATYGKLTLKLL
CTTGKLPVPWPTLVTTLGYGVQCFARYPDHMKQHDFFKSAMPEGYVQERTIFFKDDGNYKTRAEVKFEGDTLVNRIELKGIDFKED
GNILGHKLEYNYNSHNVYITADKQKNGIKANFKIRHNIEDGGVQLADHYQQNTPIGDGPVLLPDNHYLSYQSALFKDPNEKRDHMV
LLEFLTAAGITEGMNELYKHHHHHH

Amino acid sequence of *Danio* C-H-Y:

MSKGEELFGGIVPILVELEGDVNGHKFSVSGEGEGDATYGKLTLKFICTTGKLPVPWPTLVTTLTWGVQCFSRYPDHMKQHDFFKSV
MPEGYVQERTIFFKDDGNYKTRAEVKFEGDTLVNRIELKGIDFKEDGNILGHKLEYNYISHNVYITADKQKNGIKANFKARHNITDGS
VQLADHYQQNTPIGDGPVILPDNHYLSTQSALSKDPNEKRDHMVLLEFVTAAGITHGMDELKGSGGSRVHGG**C**FPGSALVSLQDG
GQKAVKDLNPGDKVLAADSAGNLVFSDFIMFT**D**RDSTTRRVFYVIETQEPVEKITLTAAHLLFVLDNSTEDLHTMTAAYASSVRAGQ
KVMVVDDSGQLKSVIVQRIYTEEQRGSFAPVTAHGTIVVDRILASCYAVIEDQGLAHLAFAPARLYYYVSSFLFPQNSSSRSNATLQQE
GVHWYSRLLYQMGTWLLDSNMLHPLGMSVNSSGGLQGSGGSMSKGEELFTGVVPILVELDGDVNGHKFSVSGEGEGDATYGKL
TLKLLCTTGKLPVPWPTLVTTLGYGVQCFARYPDHMKQHDFFKSAMPEGYVQERTIFFKDDGNYKTRAEVKFEGDTLVNRIELKGID
FKEDGNILGHKLEYNYNSHNVYITADKQKNGIKANFKIRHNIEDGGVQLADHYQQNTPIGDGPVLLPDNHYLSYQSALFKDPNEKRD
HMVLLEFLTAAGITEGMNELYKHHHHHH

Amino acid sequence of *Xenopus* C-H-Y:

MSKGEELFGGIVPILVELEGDVNGHKFSVSGEGEGDATYGKLTLKFICTTGKLPVPWPTLVTTLTWGVQCFSRYPDHMKQHDFFKSV
MPEGYVQERTIFFKDDGNYKTRAEVKFEGDTLVNRIELKGIDFKEDGNILGHKLEYNYISHNVYITADKQKNGIKANFKARHNITDGS
VQLADHYQQNTPIGDGPVILPDNHYLSTQSALSKDPNEKRDHMVLLEFVTAAGITHGMDELYKGSGGSRSGG**C**FPAGARVMVEFG
GTKAVKDLRPGDRVLSSDPQGNLLYSDFLMFI**D**QERDVKKLFYVIETSQRKIRLTAAHLLFVAQTKVNGTRSFKSVFASNIQPGDLIYT
ADPKTMTLKAVKVEKVDLEEDTGAYAPLTAHGTVVIDQVLASCYAVIEEHTWAHLAFAPLRFGMSLSSYIYPRDSSPPSGLQPHHQV
DLQSHHQVDLQSHHQVDLQSHHQLEGIHWYSQLLYQIGTWLLDSNSLHPLGMATKSSLQGSGGSMSKGEELFTGVVPILVELDGD
VNGHKFSVSGEGEGDATYGKLTLKLLCTTGKLPVPWPTLVTTLGYGVQCFARYPDHMKQHDFFKSAMPEGYVQERTIFFKDDGNY
KTRAEVKFEGDTLVNRIELKGIDFKEDGNILGHKLEYNYNSHNVYITADKQKNGIKANFKIRHNIEDGGVQLADHYQQNTPIGDGPVL
LPDNHYLSYQSALFKDPNEKRDHMVLLEFLTAAGITEGMNELYKHHHHHH

Figure S2. Amino acid sequences of *Drosophila*, *Danio*, and *Xenopus* C-H-Y. Cyan fluorescent protein (cyan); SHhC (gray); yellow fluorescent protein (yellow). The catalytically essential C1 and D46 residues, are labeled in bold. Sequences were verified by whole plasmid sequencing (Azenta).

bioRxiv preprint doi: https://doi.org/10.64898/2026.03.09.710561; this version posted March 11, 2026. The copyright holder for this preprint (which was not certified by peer review) is the author/funder, who has granted bioRxiv a license to display the preprint in perpetuity. It is made available under aCC-BY 4.0 International license.

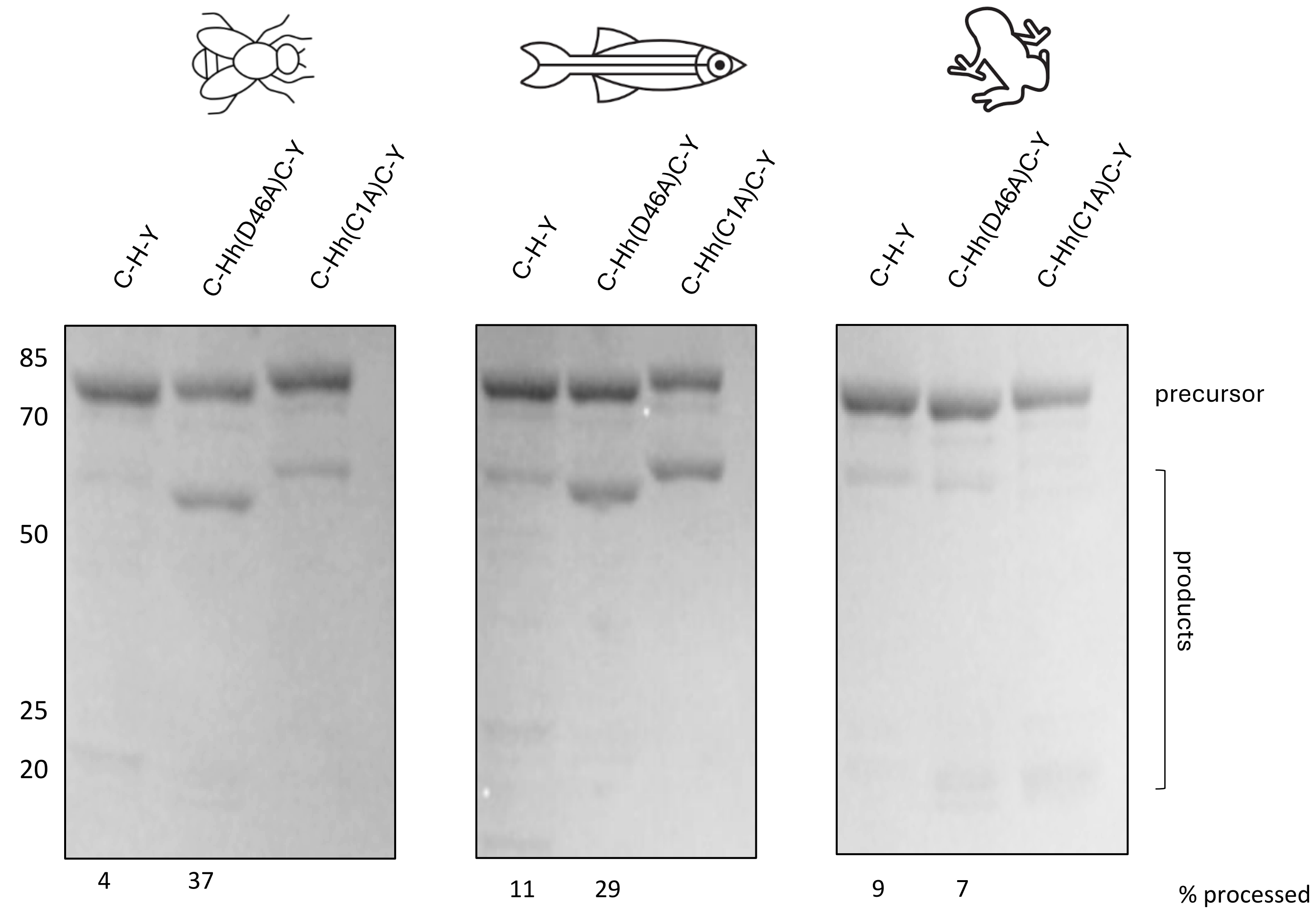

Figure S3. SDS-PAGE of bacterially expressed, Ni-NTA purified Dme, Dre, Xla C-H-Y reporter constructs (WT, D46A, C1A). The extent of precursor autoprocessing that occurred during the expression and purification is indicated below each gel. Protein samples were run at 2 µM precursor concentration, and the extent of processing was determined by ImageJ.

bioRxiv preprint doi: https://doi.org/10.64898/2026.03.09.710561; this version posted March 11, 2026. The copyright holder for this preprint (which was not certified by peer review) is the author/funder, who has granted bioRxiv a license to display the preprint in perpetuity. It is made available under aCC-BY 4.0 International license.

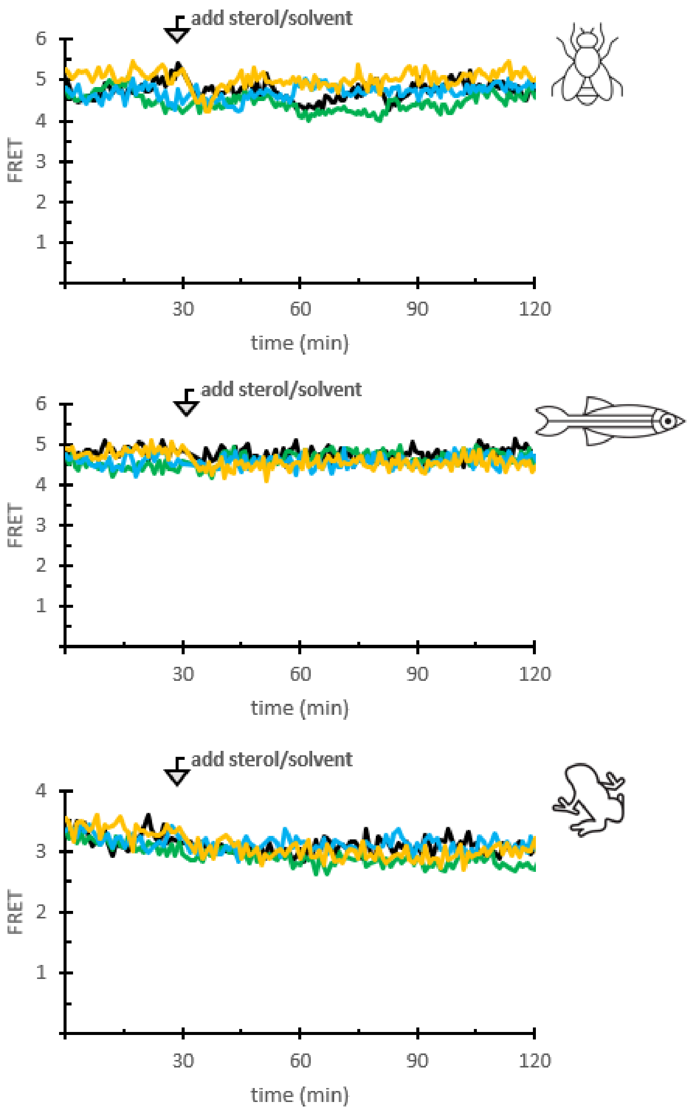

Figure S4. Alanine point mutation at the catalytically essential C1 residue of SHhC eliminates activity. Reactions were run for 2 hours at 30ºC in 96-wellplates using 0.2µM C-Hh(C1A)C-Y, Bis-Tris buffer (pH 7.1) containing EDTA (5 mM) and NaCl (0.5 M), and Fos-choline 12 (1.5 mM), with either no sterol (black); 50µM cholesterol (green); 2-ACC (cyan); or 100mM DTT (yellow).

bioRxiv preprint doi: https://doi.org/10.64898/2026.03.09.710561; this version posted March 11, 2026. The copyright holder for this preprint (which was not certified by peer review) is the author/funder, who has granted bioRxiv a license to display the preprint in perpetuity. It is made available under aCC-BY 4.0 International license.

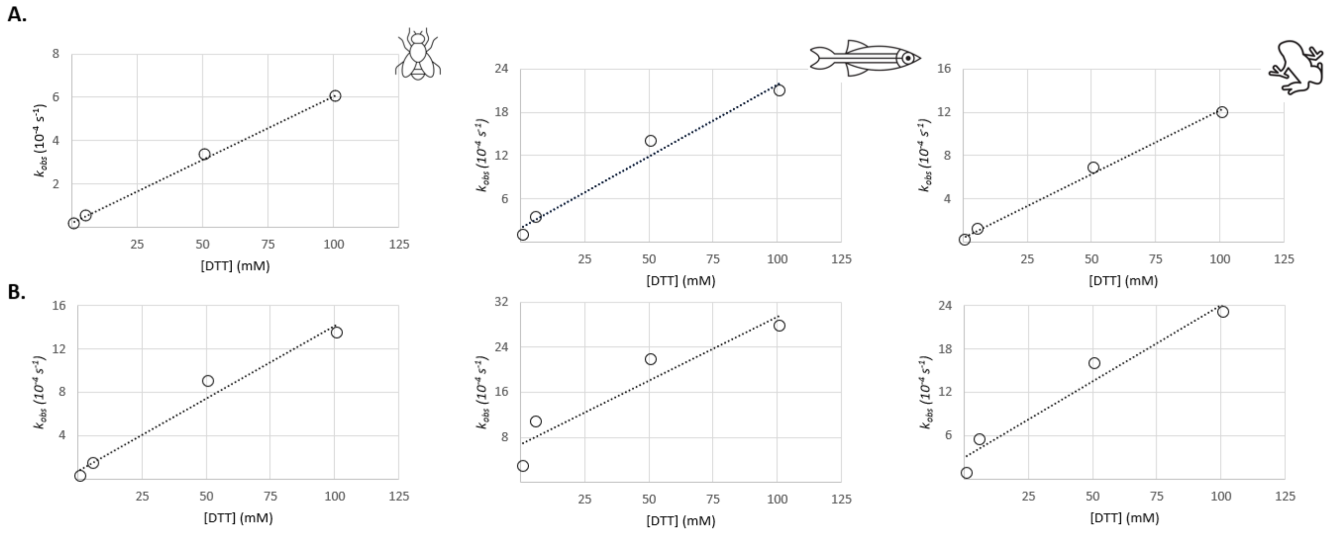

Figure S5. Rates of thioloysis (k_{obs}) plotted as a function of increasing DTT concentration. Reactions were run for 2 hours at 30ºC in 96-well plates using 0.2 µM C-H-Y (*A*) or C-H(D46A)-Y (*B*), Bis-Tris buffer (pH 7.1) also containing EDTA (5mM) and NaCl (0.5M), and Fos-choline 12 (1.5mM). (*A*) DTT concentration vs. k_{obs} of DTT-based processing for WT constructs from Dme (left), Dre (middle), or Xla (right). (*B*) DTT concentration vs. k_{obs} as an (A), expect for (D46A) mutants.

bioRxiv preprint doi: https://doi.org/10.64898/2026.03.09.710561; this version posted March 11, 2026. The copyright holder for this preprint (which was not certified by peer review) is the author/funder, who has granted bioRxiv a license to display the preprint in perpetuity. It is made available under aCC-BY 4.0 International license.

	1	2	3	4	5	6	7	8	9	10	11	12
A	14:0 Lyso PG (I)	16:0 Lyso PG (I)	18:0 Lyso PG (I)	18:1 Lyso PG (I)	CTAB (I)	BAM (I)	DTAC (I)	Sodium cholate hydrate (I)	Deoxycholic acid sodium salt (I)	MSDH (I)	N-Laurylsarcosine sodium salt (I)	Sodium dodecyl sulfate (I)
B	Lithium dodecyl sulfate (I)	APO 8 (N)	APO 9 (N)	Apo 10 (N)	APO 11 (N)	APO 12 (N)	C_8E_4 (N)	C_8E_5 (N)	$C_{12}E_8$ (N)	$C_{12}E_9$ (N)	Brij 35 (N)	Brij 56 (N)
C	Brij 58 (N)	Genapol X-080 (N)	Facade-EM (N)	Facade-EPC (N)	Facade-TEG (N)	Facade-TEM (N)	Facade-TFA1 (N)	MEGA-8 (N)	MEGA-9 (N)	MEGA-10 (N)	Mal(11.1) (N)	Mal(11.2) (N)
D	DDAO (N)	n-Decyl-β-D-maltoside (N)	n-Dodecyl-β-D-maltoside (N)	n-Hexadecyl-β-D-maltoside (N)	n-Tetradecyl-β-D-maltoside (N)	n-Tridecyl-β-D-maltoside (N)	n-Undecyl-β-D-maltoside (N)	HECAMEG (N)	IPTG (N)	n-Decanoylsucrose (N)	N-Nonyl-β-D-glucoside (N)	N-Octyl-β-D-glucoside (N)
E	OSGP (N)	Pluronic F-68 (N)	Pluronic F-127 (N)	Sucrose monolaurate (N)	Thesit (N)	Triton X-100 (N)	Triton X-114 (N)	Nonylphenyl polyethylene glycol (N)	Tween 20 (N)	Tween 80 (N)	GDN (N)	CHAPS (Z)
F	CHAPSO (Z)	BIG CHAP (Z)	Deoxy BIG CHAP (Z)	DDMAB (Z)	LDAO (Z)	Sulfobetaine 8 (Z)	Sulfobetaine 10 (Zwittergent 3-10) (Z)	Sulfobetaine 12 (Zwittergent 3-12) (Z)	Sulfobetaine 14 (Zwittergent 3-14) (Z)	Sulfobetaine 16 (Zwittergent 3-16) (Z)	06:0 PC (DHPC) (Z)	07:0 PC (DHPC) (Z)
G	06:0 Lyso PC (Z)	07:0 Lyso PC (Z)	08:0 Lyso PC (Z)	09:0 Lyso PC (Z)	10:0 Lyso PC (Z)	11:0 Lyso PC (Z)	12:0 Lyso PC (Z)	13:0 Lyso PC (Z)	14:0 Lyso PC (Z)	15:0 Lyso PC (Z)	16:0 Lyso PC (Z)	17:0 Lyso PC (Z)
H	18:0 Lyso PC (Z)	18:1 Lyso PC (Z)	MAPCHO-10 (FOS-Choline-10) (Z)	MAPCHO-12 (FOS-Choline-12) (Z)	MAPCHO-14 (FOS-Choline-14) (Z)	MAPCHO-16 (FOS-Choline-16) (Z)	NDSB-195 (NS)	NDSB-201 (NS)	NDSB-211 (NS)	NDSB-221 (NS)	NDSB-256 (NS)	NDSB-256-4T (NS)

Figure S6. Key for the 96-well detergent screen. This screen from Hampton Research consisted of 13 ionic (I, *wells* **A01-B01**) detergents, 46 non-ionic (N, *wells* **B02-E11**) detergents, 31 zwitterionic detergents (Z, *wells* **E12-H06**), and 6 non-detergent Sulfobetaines (NS, *wells* **H07-H12**). For the ionic detergents, there were 4 cationic (I, *wells* **A05, A06, A07, and A10**), and 9 anionic (I, *wells* **A01-A04, A08, A09, A10-B01**).

bioRxiv preprint doi: https://doi.org/10.64898/2026.03.09.710561; this version posted March 11, 2026. The copyright holder for this preprint (which was not certified by peer review) is the author/funder, who has granted bioRxiv a license to display the preprint in perpetuity. It is made available under aCC-BY 4.0 International license.

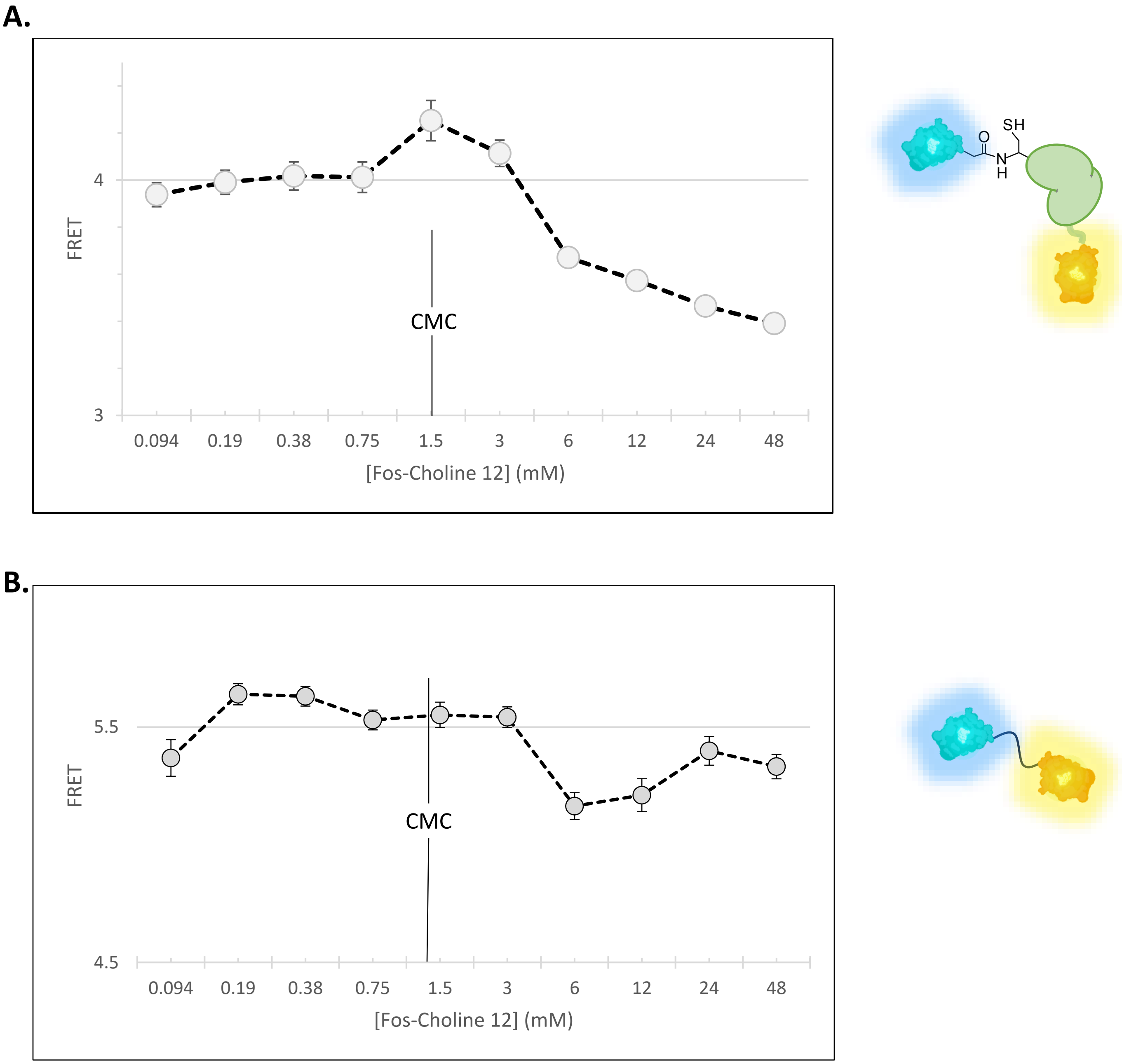

Figure S7. Impact of Fos-Choline 12 concentration on FRET signal from C-H-Y. (*A*) Concentration-response plot of the average FRET for Wild-Type *Drosophila* C-H-Y as a function of increasing Fos-Choline 12. (*B*) Concentration-dependent traces representing the average FRET level recorded from control construct, C-Y, at the same Fos-Choline 12 concentration as in (A).

bioRxiv preprint doi: https://doi.org/10.64898/2026.03.09.710561; this version posted March 11, 2026. The copyright holder for this preprint (which was not certified by peer review) is the author/funder, who has granted bioRxiv a license to display the preprint in perpetuity. It is made available under aCC-BY 4.0 International license.

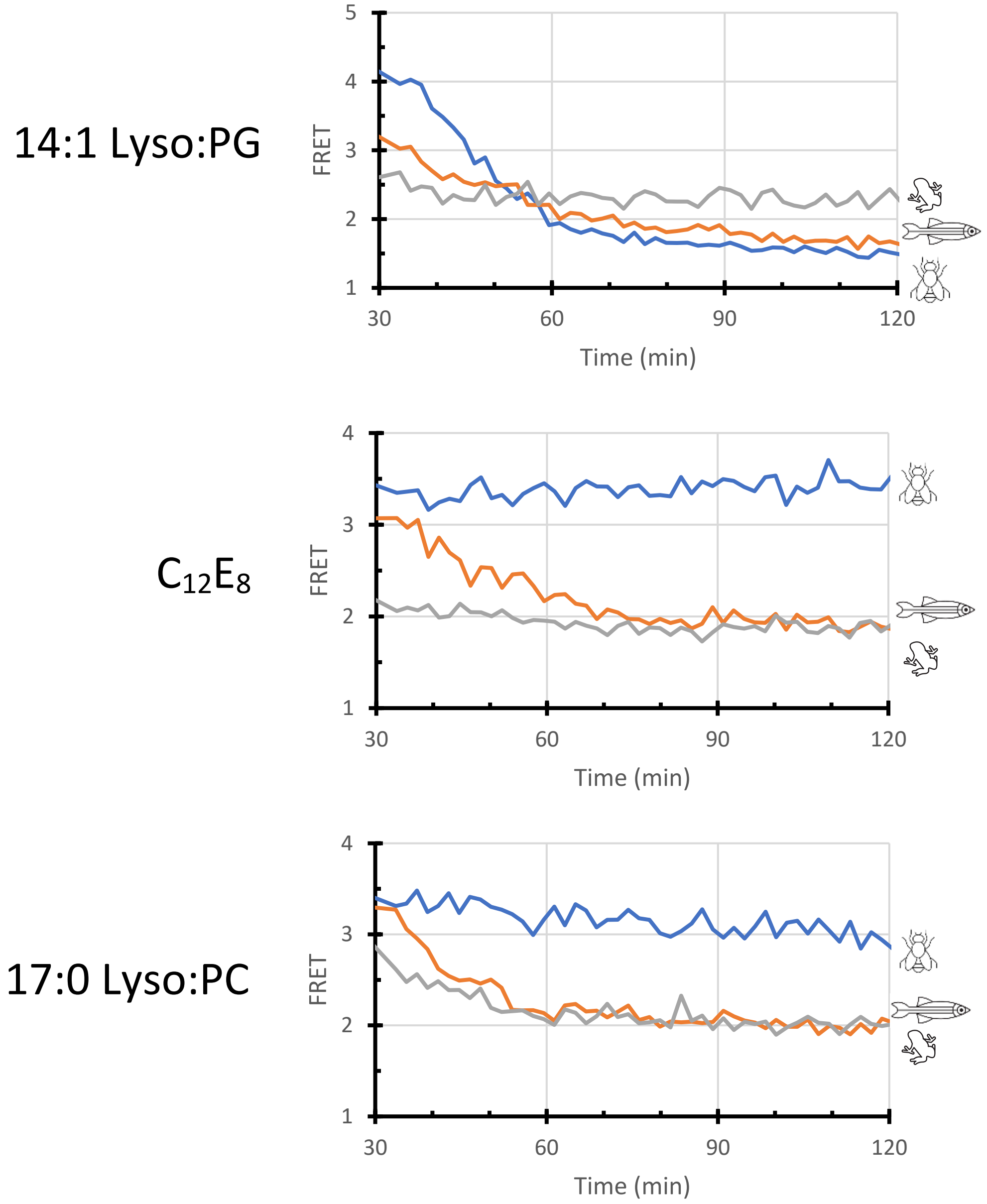

Figure S8. Species specific detergent effects. Kinetic traces for C-H-Y cholesterolysis using either 14:1 Lyso:PG (*well* **A1**, top), $C_{12}E_8$ (*well* **B9**, middle), or 17:0 Lyso PC (*well* **G12**, bottom) in place of Fos-choline 12. (top) 14:1 Lyso:PG resulted in rapid cholesterolysis for both *Drosophila* (blue) and *Danio* (orange), but no cholesterolysis activity was apparent for *Xenopus* (gray). (middle) $C_{12}E_8$ resulted in moderate cholesterolysis for *Danio* (orange), but no activity for both *Drosophila* (blue) and *Xenopus* (gray). (bottom) 17:0 Lyso:PC supported rapid cholesterolysis for *Xenopus* (gray), moderate cholesterolysis for *Danio* (orange), and little to no cholesterolysis activity for *Drosophila* (blue). Samples were monitored for 2 hours at 30°C in 96-wellplates using 0.2 µM C-H-Y in Bis-Tris buffer (pH 7.1) with EDTA (5 mM), NaCl (0.5 M), with detergent at the respective CMC.

bioRxiv preprint doi: https://doi.org/10.64898/2026.03.09.710561; this version posted March 11, 2026. The copyright holder for this preprint (which was not certified by peer review) is the author/funder, who has granted bioRxiv a license to display the preprint in perpetuity. It is made available under aCC-BY 4.0 International license.

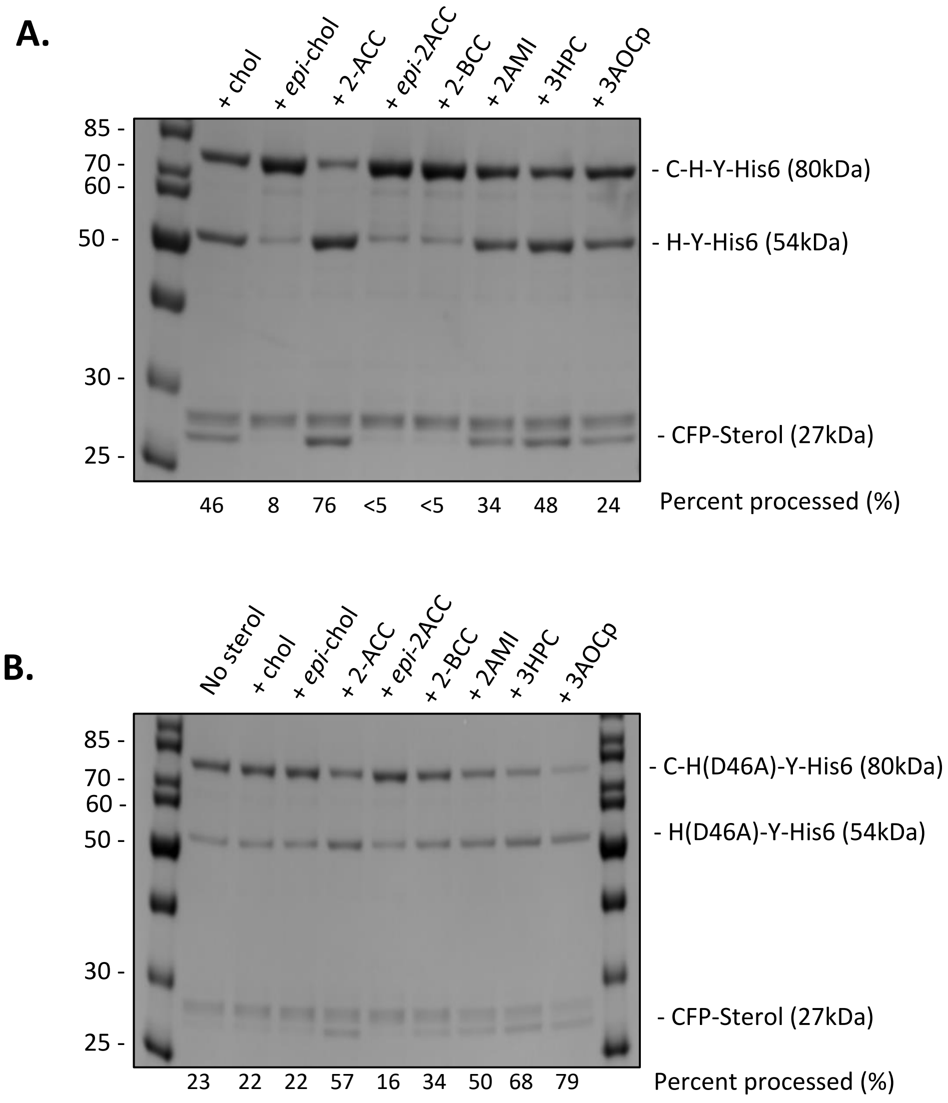

Figure S9. SDS-PAGE Analysis of substrate activity for chemical rescue sterols with *Drosophila* Wild-Type C-H-Y and mutant C-H(D46A)-Y. Reactions were run for 3 hours at 30°C using 1 µM protein, Bis-Tris buffer (pH 7.1) also containing EDTA (5mM) and NaCl (0.5 M), and Fos-choline 12 (1.5 mM). (*A*) SDS-PAGE of *Drosophila* Wild-Type C-H-Y protein with 50 µM of sterol added for each, except 2-BCC which contained 100 µM of sterol. (B) SDS-PAGE of *Drosophila* mutant C-H(D46A)-Y protein with 50 µM of sterol added for each, except 2-BCC which contained 100 µM of sterol. Percent processed values used ImageJ to compare the amount of precursor between the samples (A), or the total percent processing from comparing the product bands to the total band area (B).

bioRxiv preprint doi: https://doi.org/10.64898/2026.03.09.710561; this version posted March 11, 2026. The copyright holder for this preprint (which was not certified by peer review) is the author/funder, who has granted bioRxiv a license to display the preprint in perpetuity. It is made available under aCC-BY 4.0 International license.

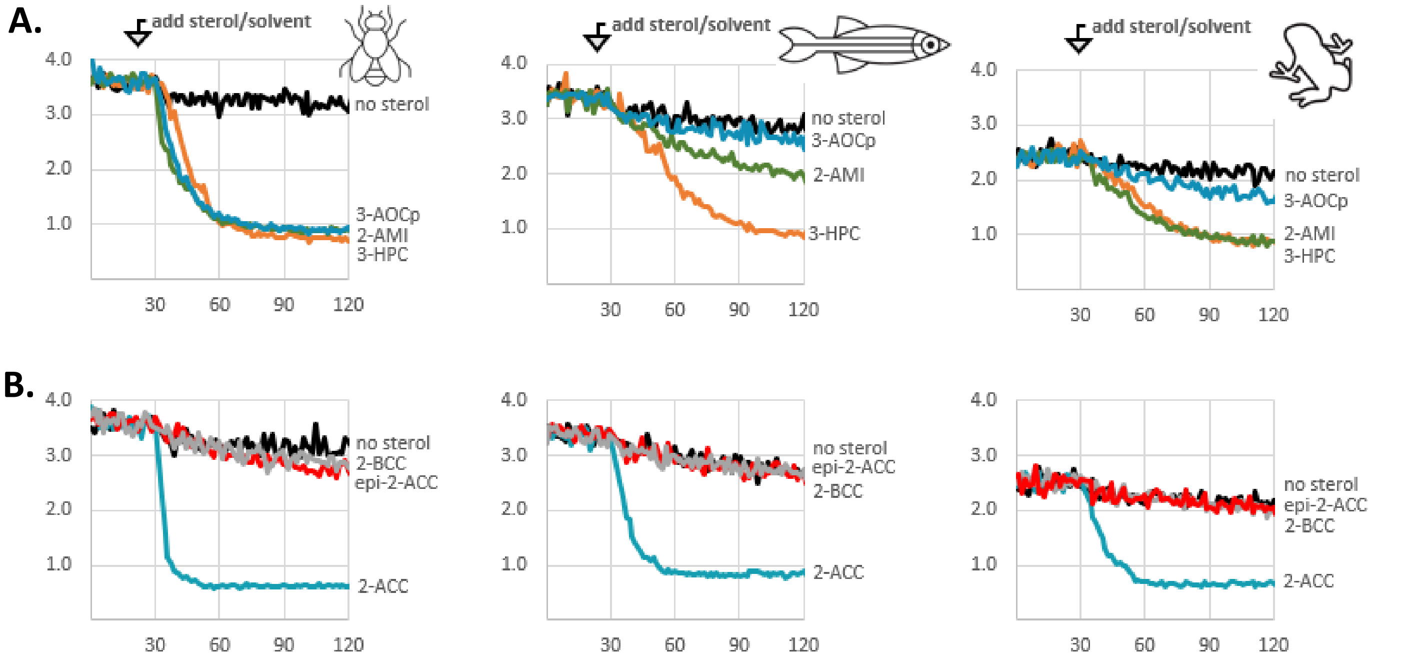

Figure S10. Substrate activity of synthetic sterols using Wild-Type C-H-Y reporter constructs. Samples were monitored for 2 hours at 30ºC in 96-wellplates using 0.2 µM C-H-Y in Bis-Tris buffer (pH 7.1) with EDTA (5 mM), NaCl (0.5 M), and Fos-choline 12 (1.5 mM). (*A*) Kinetic traces of *Drosophila* (left), *Danio* (middle), and *Xenopus* (right) C-H-Y with either no sterol (black) or with 50 µM 2-AMI (green), 3-HPC (orange), or 3-AOCp (blue). (*B*) Kinetic traces of *Drosophila* (left), *Danio* (middle), and *Xenopus* (right) C-H-Y with either no sterol (black) or with 50 µM 2-ACC (cyan), 2-BCC (gray), and *epi* 2-ACC (red).

bioRxiv preprint doi: https://doi.org/10.64898/2026.03.09.710561; this version posted March 11, 2026. The copyright holder for this preprint (which was not certified by peer review) is the author/funder, who has granted bioRxiv a license to display the preprint in perpetuity. It is made available under aCC-BY 4.0 International license.

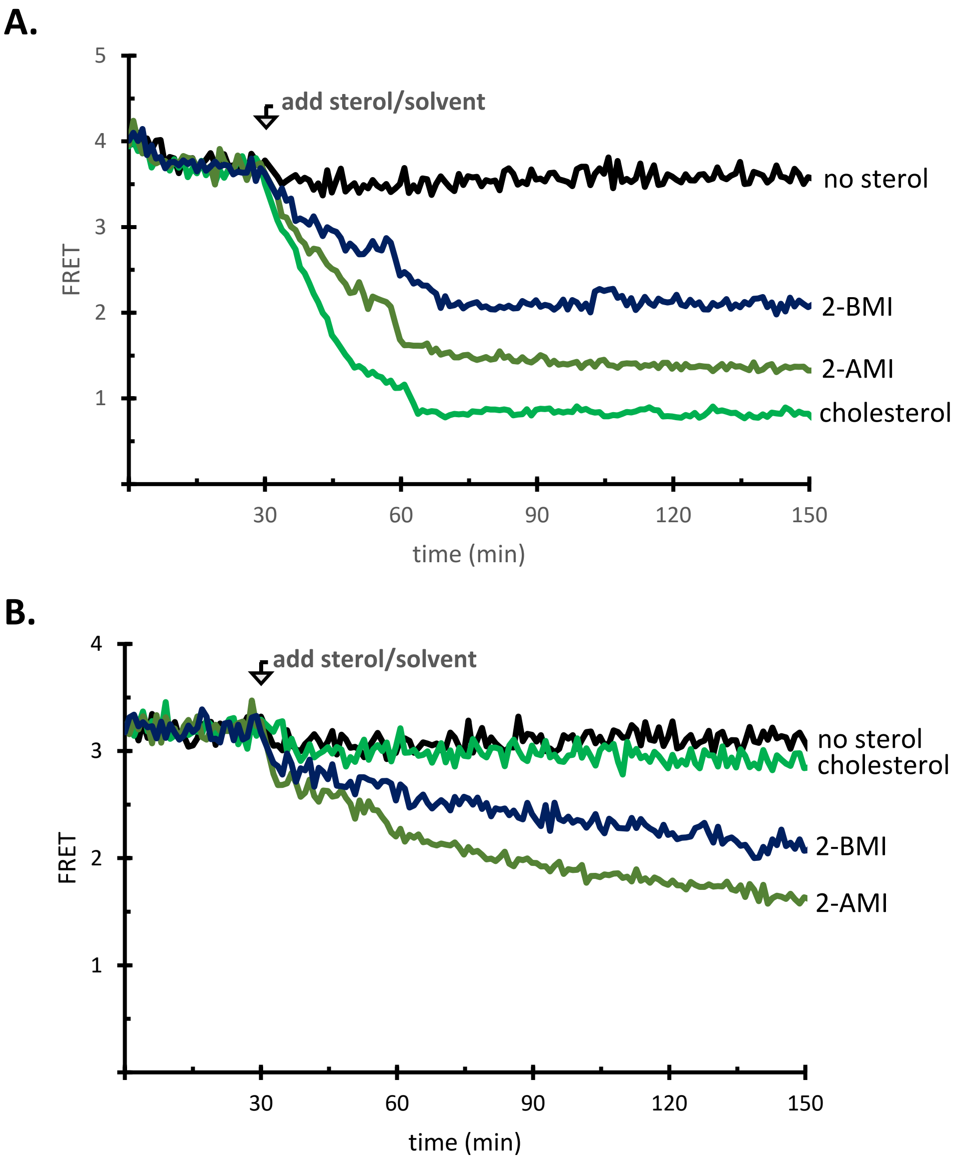

Figure S11. Substrate activity of cholestanol 2-imidazolmethyl epimers with *Drosophila* Wild-Type C-H-Y and mutant C-H(D46A)-Y. Reactions were monitored for 2.5 hours at 30ºC in 96-wellplates using 0.2 µM reporter protein in Bis-Tris buffer (pH 7.1) with EDTA (5 mM) and NaCl (0.5 M), and Fos-choline 12 (1.5 mM). (*A*) Kinetic trace of *Drosophila* Wild-Type C-H-Y with either no sterol (black) or with 50 µM cholesterol (light green), 2-AMI (Dark green), or 2-BMI (Blue). (*B*) Kinetic trace of *Drosophila* mutant C-H(D46A)-Y with either no sterol (black) or with 50µM cholesterol (light green), 2-AMI (Dark green), or 2-BMI (Blue).

bioRxiv preprint doi: https://doi.org/10.64898/2026.03.09.710561; this version posted March 11, 2026. The copyright holder for this preprint (which was not certified by peer review) is the author/funder, who has granted bioRxiv a license to display the preprint in perpetuity. It is made available under aCC-BY 4.0 International license.

Supporting Methods

Construction of FRET reporter constructs for Xla and Dru SHhC. Gene fragments encoding SHhC with an upstream SHhN peptide were synthesized with 5' (Xho I) and 3' (Pst I) sites, codon-optimized for *E. coli* expression, and cloned into a modified pBAD33 FRET expression vector, described previously.[1, 2] Reporter constructs for wild type, C1A and D46A SHhC were prepared in this same way. Whole plasmid sequencing (Plasmid-EZ, Azenta) was used to confirm construct assembly. The expressed C-H-Y polyprotein carries a C-terminal His-tag for purification.

***E. coli* expression and Ni-NTA purification of C-H-Y FRET reporter**: E. coli strain LMG194 was used mainly for expressing C-H-Y. Following previous work,[3, 4] a swath of single colonies of the C-H-Y expression strain was transferred from an LB-agar plate containing 50 µg/mL chloramphenicol (CAM) into a 125 mL baffled flask containing 50 mL of LB with CAM (50 µg/mL). The flask was incubated at 37 ^{o}C with shaking (225 RPM) until the optical density reading (OD_{600}) reached between 0.6-0.8 units. Protein expression was then induced by adding arabinose (0.2 % w/v) and the flask was incubated for additional 18-22 hours at reduced temperature (16 ^{o}C). After the induction period, the broth was transferred to 50 mL conical tube and centrifuged at 10,000 RPM for 10 minutes at 4 ^{o}C. The broth was discarded and the pellet resuspended in 1 mL of lysis buffer (20mM Na_2HPO_4 buffer pH 7, with 400mM NaCl, 100mM KCl, 10mM imidazole, 0.5% Triton X-100, and 10% glycerol). Lysozyme was added to the homogenate (0.2mg / mL, final) and incubated for 10 minutes at room temperature, followed by two freeze-thaw cycles at -80 C. To the viscous mixture, we then added 10 µL of Longlife DNase (G-Biosciences) plus 5 µL of mung bean nuclease (NEB) and incubated at room temperature with gentle mixing for 15 minutes. All steps past this point were done on ice with pre-chilled buffers. Once the total lysate was free flowing, it was combined with an equal volume of 2x binding buffer (40mM Na_2HPO_4 pH 7, 1M NaCl, 20mM imidazole, and 20% glycerol). After a final vortex, insoluble cellular debris was removed by centrifugation (14,000 RPM, 15 min). His-tagged C-H-Y was purified from this soluble fraction using a His SpinTrap column (Cytiva). After passing 500 µl of wash buffer 1 (500mM NaCl, 20mM Na_2HPO_4, 37.5mM imidazole and 10% glycerol), and 500 µl of wash buffer 2 (500mM NaCl, 20mM Na_2HPO_4, 75mM imidazole and 10% glycerol), the reporter protein was eluted with 300 µL of elution buffer (500mM NaCl, 20mM Na_2HPO_4, 500 mM imidazole and 10% glycerol). To protect cysteine residues of SHhC, 3 µL of TCEP (500mM) was added to the elution and stored at -80 C. Concentration and purity of C-H-Y in the elution was determined by SDS-PAGE using BioRad Gel Doc EZ system.

FRET assays for SHhC cholesterolysis: Activity measurements were conducted in 100 ul volume with C-H-Y between 100-200 nM, along with Bis-Tris propane buffer (50 mM, pH 7.2), Ethylenediaminetetraacetic acid (EDTA) (0.5 mM) and NaCl (100 mM). Unless stated otherwise, we used Fos-choline 12 (1.5 mM, final) to solubilize cholesterol and TCEP (5 mM, final) as a reducing agent. Assays were conducted at 30 ^{o}C in 96 well NBS™ Microplates (Corning) using a

bioRxiv preprint doi: https://doi.org/10.64898/2026.03.09.710561; this version posted March 11, 2026. The copyright holder for this preprint (which was not certified by peer review) is the author/funder, who has granted bioRxiv a license to display the preprint in perpetuity. It is made available under aCC-BY 4.0 International license.

BioTek Synergy H1 plate reader with Gen5 software. Sample FRET readings were recorded every 1-2 minutes as the 540nm/460nm emission ratio after excitation at 460 nm. Reactions were initiated by addition of cholesterol from an ethanol stock.

Determination of k_{max} and K_M values from FRET assay data:

For each C-H-Y/sterol, the maximum rate of sterolysis (k_{max}) was estimated from the experimental FRET loss data at the highest sterol concentration by curve fitting to a first order exponential decay.

$$FRET_t = A*e^{(-kobs*FRET)} + C$$

K_M values for substrate sterols in the C-H-Y system was estimated by plotting the initial rate of FRET loss (slope of experimental data) as a function of increasing initial substrate concentration. Curve fitting to a Michaelis-Menten equation was carried out using GraphPad prism and Excel.

Chemical Rescue: Kinetic experiments were carried using the FRET approach (as above) except that chemically modified cholestanol derivatives replaced cholesterol as potential substrates. FRET assays included six replicates for each C-H-Y construct (WT and D46A) and each cholestanol derivative. Selected compounds, 2-ACC and 2-BCC, were tested further in concentration-response experiments (as above) to determine K_M value.

Detergent screening for compatibility with SHhC cholesterolysis: Detergent screening for Dme, Xla, and Dre cholesterolysis used the 96-member library from Hampton Research (cat: HR2407). In preparation for the screen, the detergent library was thawed at room temperature, then warmed to 37°C until all compounds had fully dissolved. Once complete, 10 µL of each detergent was transferred to the 96-well assay plate, followed by the addition of 88 uL of master mix containing C-H-Y in the buffer components used in the cholesterolysis assays (above), with the exception of fos-choline 12. Prior to initiating cholesterolysis, FRET readings from each sample were recorded for 30 minutes for the purpose of isolating potential detergent effects on C-H-Y FRET signal. After that pre-incubation, cholesterolysis was initiated by adding 2 µL of 2.5 mM cholesterol stock to each well and FRET was monitored for another 1.5 hours. Reaction progress curves for FRET loss were analyzed for selected wells to obtain the apparent k_{max} values.

Thiolysis of SHhC precursor using the FRET reporter: C-H-Y thiolysis assays were carried out in a manner similar to the cholesterolysis experiments except that here the reactions were initiated with dithiothreitol (DTT) added from aqueous stock in Bis-Tris buffer. First order exponential decay curves were fit to experimental FRET-loss data to determine k_{DTT} values.

bioRxiv preprint doi: https://doi.org/10.64898/2026.03.09.710561; this version posted March 11, 2026. The copyright holder for this preprint (which was not certified by peer review) is the author/funder, who has granted bioRxiv a license to display the preprint in perpetuity. It is made available under aCC-BY 4.0 International license.

General Procedures. NMR spectra were acquired using a Bruker Avance III 600 MHz or 800 MHz spectrometer at 25 °C. Calibration was by the residual solvent signal ($CDCl_3$: ^1H = 7.26 ppm. ^{13}C = 77.0 ppm). Preparative TLC was performed on glass-backed plates (10 cm in length) coated with a 0.25 mm layer of silica gel 60 F254.

Synthesis of 2-carboxycholestanol isomers

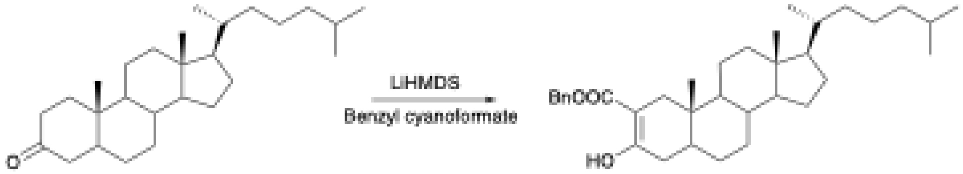

A solution of 5α-cholestan-3-one in anhydrous THF (75 mg/mL) under N_2 at -78 °C was treated with LiHMDS (1.25 M in THF, 1.3 eq), and the solution was stirred for 20 min., followed by the addition of benzyl cyanoformate (1.25 eq.).[5] The reaction was allowed to warm to rt over two hours, then poured into 30 mL of 5% conc. HCl and extracted with 3 x 15 mL of hexanes/EtOAc 4:1. The combined organic layers were washed with 10% conc. HCl and brine, then dried over Na_2SO_4 and concentrated *in vacuo* to yield the product. Minor *O*-acyl and 4-acyl byproducts visible by NMR could not be separated, and the crude product (80% pure by NMR) was used in the next step without further purification.

2-(Benzyloxycarbonyl)-5α-cholestan-3-one (enol form): ^1H NMR (600 MHz, $CDCl_3$): 12.09 (1H, s), 7.40-7.30 (5H, m), 5.203 (2H, dd, *J* = 12.6, 38.1 Hz), 2.353 (1H, d, *J* = 15.8 Hz), 0.900 (3H, d, *J* = 6.5Hz), 0.864 (3H, d, *J* = 6.6 Hz), 0.860 (3H, d, *J* = 6.6 Hz), 0.747 (3H, s), 0.658 (3H, s).

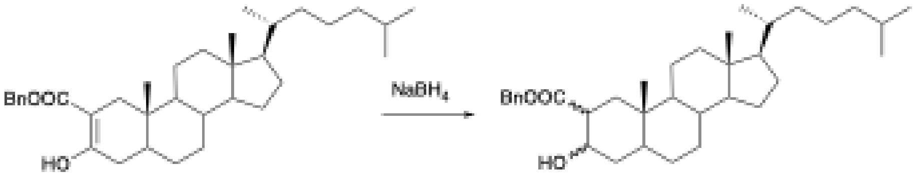

To a solution of the β-keto ester in 2:1 DCM/MeOH (9 mg/mL) was added $NaBH_4$ (4 eq) in one portion. The reaction was monitored by TLC, and when no starting material was visible, the reaction was quenched with 5% conc. HCl. The contents were extracted thoroughly with hexanes/EtOAc 4:1, and the combined organic layers were washed with brine, dried over Na_2SO_4, and concentrated *in vacuo* to yield the crude product which was purified by preparative TLC (hexanes/EtOAc 4:1).

bioRxiv preprint doi: https://doi.org/10.64898/2026.03.09.710561; this version posted March 11, 2026. The copyright holder for this preprint (which was not certified by peer review) is the author/funder, who has granted bioRxiv a license to display the preprint in perpetuity. It is made available under aCC-BY 4.0 International license.

(2α,3α)-2-Benzyloxycarbonylcholestan-3-ol (24%). ^{1}H NMR (600 MHz, $CDCl_3$): 7.40-7.31 (5H, m), 5.145 (2H, dd, *J* = 12.3, 29.9 Hz), 4.236 (1H, m), 3.088 (1H, br s), 2.615 (1H, ddd, *J* = 2.3, 3.5, 13.4 Hz), 0.893 (3H, d, *J* = 6.5 Hz), 0.863 (3H, d, *J* = 6.6 Hz), 0.859 (3H, d, *J* = 6.6 Hz), 0.799 (3H, s), 0.643 (3H, s).

(2β,3α)-2-Benzyloxycarbonylcholestan-3-ol (10%). ^{1}H NMR (600 MHz, $CDCl_3$): 7.40-7.31 (5H, m), 5.131 (2H, dd, *J* = 12.3, 19.3 Hz), 4.063 (1H, m), 3.490 (1H, br s), 2.512 (1H, dd, *J* = 1.7, 12.2 Hz), 0.893 (3H, d, *J* = 6.5 Hz), 0.863 (3H, d, *J* = 6.6 Hz), 0.859 (3H, d, *J* = 6.6 Hz), 0.811 (3H, s), 0.636 (3H, s).

(2α,3β)-2-Benzyloxycarbonylcholestan-3-ol (36%). ^{1}H NMR (600 MHz, $CDCl_3$): 7.40-7.31 (5H, m), 5.156 (2H, dd, *J* = 12.4, 18.4 Hz), 3.836 (1H, td, *J* = 4.9, 10.7 Hz), 2.614 (1H, br s), 2.529 (1H, ddd, *J* = 3.8, 10.3, 13.1 Hz), 0.892 (3H, d, *J* = 6.5 Hz), 0.862 (3H, d, *J* = 6.6 Hz), 0.858 (3H, d, *J* = 6.6 Hz), 0.839 (3H, s), 0.641 (3H, s).

(2β,3β)-2-Benzyloxycarbonylcholestan-3-ol (30%). ^{1}H NMR (600 MHz, $CDCl_3$): 7.41-7.33 (5H, m), 5.193 (1H, d, *J* = 12.3 Hz), 5.061 (1H, d, *J* = 12.3 Hz), 3.693 (1H, d, *J* = 11.8 Hz), 3.606 (1H, tt, *J* = 4.8, 11.8 Hz), 2.933 (1H, t, *J* = 5.9 Hz), 2.400 (1H, dd, *J* = 2.3, 14.0 Hz), 1.951 (1H, dt, *J* = 3.3, 12.6 Hz), 0.891 (3H, d, *J* = 6.5 Hz), 0.861 (3H, d, *J* = 6.6 Hz), 0.857 (3H, d, *J* = 6.6 Hz), 0.646 (3H, s), 0.622 (3H, s).

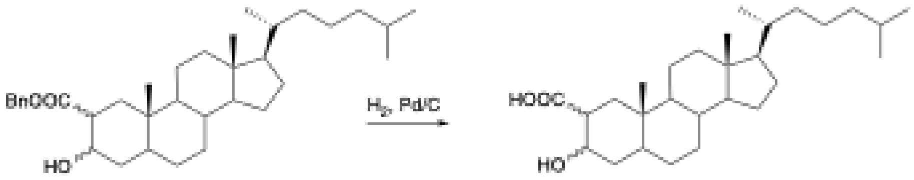

To a solution of each hydroxy ester in EtOAc (5 mg/mL) was added 10% Pd/C (2 mg/mL) and the mixture was stirred under H_2 (1 atm) for two hrs. $CHCl_3$ was added to dissolve the solid products that precipitated during the course of the reaction, followed by filtration through celite to remove the catalyst. Concentration *in vacuo* afforded the deprotected hydroxy acids in quantitative yield.

(2α,3α)-2-Carboxycholestan-3-ol (*epi* 2-ACC). ^{1}H NMR (600 MHz, $CDCl_3$): 4.272 (1H, m), 2.632 (1H, d, *J* = 13.0 Hz), 0.898 (3H, d, *J* = 6.5 Hz), 0.863 (3H, d, *J* = 6.6 Hz), 0.859 (3H, d, *J* = 6.6 Hz), 0.823 (3H, s), 0.651 (3H, s).

(2β,3α)-2-Carboxycholestan-3-ol (*epi* 2-BCC). ^{1}H NMR (600 MHz, $CDCl_3$): 4.165 (1H, m), 2.519 (1H, d, *J* = 12.1 Hz), 0.897 (3H, d, *J* = 6.5 Hz), 0.864 (3H, d, *J* = 6.6 Hz), 0.859 (3H, d, *J* = 6.6 Hz), 0.839 (3H, s), 0.649 (3H, s).

bioRxiv preprint doi: https://doi.org/10.64898/2026.03.09.710561; this version posted March 11, 2026. The copyright holder for this preprint (which was not certified by peer review) is the author/funder, who has granted bioRxiv a license to display the preprint in perpetuity. It is made available under aCC-BY 4.0 International license.

(2α,3β)-2-Carboxycholestan-3-ol (2-ACC). ^{1}H NMR (600 MHz, $CDCl_3$): ^{1}H NMR (800 MHz, $CDCl_3$): 3.818 (1H, td, J = 4.7, 10.6 Hz), 2.513 (1H, td, J = 3.0, 11.9 Hz), 2.048 (1H, dd, J = 3.2, 13.8 Hz), 1.965 (1H, dt, J = 3.0, 12.7 Hz), 1.809 (1H, m), 1.658 (2H, m), 1.58-1.47 (3H, m), 0.896 (3H, d, J = 6.4 Hz), 0.864 (3H, d, J = 6.6 Hz), 0.860 (3H, d, J = 6.6 Hz), 0.855 (3H, s), 0.673 (1H, td, J = 3.6, 11.4 Hz), 0.650 (3H, s). ^{13}C NMR (201 MHz): 179.57, 71.14, 56.40, 56.23, 54.03, 46.79, 44.50, 42.55, 40.05, 39.87, 39.51, 36.16, 36.07, 35.78, 35.44, 31.84, 28.26, 28.21, 28.01, 24.18, 23.82, 22.81, 22.55, 21.30, 18.67, 12.52, 12.07.

(2β,3β)-2-Carboxycholestan-3-ol (2-BCC). ^{1}H NMR (800 MHz, $CDCl_3$): 3.715 (1H, dt, J = 5.0, 11.9 Hz), 2.937 (1H, t, J = 5.7 Hz), 2.477 (1H, dd, J = 1.9, 14.1 Hz), 1.972 (1H, dt, J = 3.2, 12.8 Hz), 0.899 (3H, d, J = 6.6 Hz) 0.866 (3H, d, J = 6.6 Hz), 0.862 (3H, d, J = 6.6 Hz), 0.802 (3H, s), 0.642 (3H, s), 0.615 (1H, m). ^{13}C NMR (201 MHz): 177.89, 71.34, 56.39, 56.32, 54.64, 46.14, 43.22, 42.63, 40.01, 39.94, 39.52, 36.24, 36.17, 35.77, 35.20, 31.85, 28.20, 28.19, 28.01, 24.17, 23.84, 22.79, 22.55, 21.46, 18.68, 12.73, 12.10.

Synthesis of 2-imidazolemethylcholestanol isomers

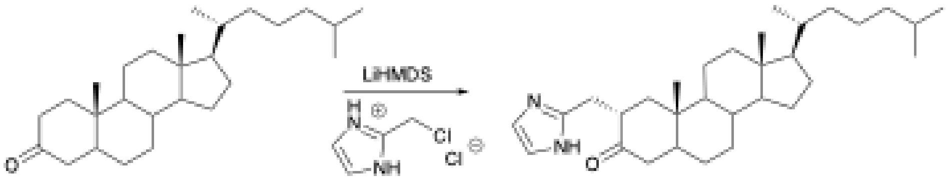

A solution of cholestan-3-one (251 mg, 0.65 mmol) in 5 mL of anhydrous THF under N_2 was cooled to -78 °C. To this was added 1.3 mL of 1 M LiHMDS/hexane (2.0 eq.) and the solution was stirred for 20 minutes at -78 °C, at which time 2-chloromethyl-1H-imidazole hydrochloride (100 mg, 1.0 eq) was added, followed by in 2 mL of anhydrous THF. The reaction was allowed to slowly warm to rt with stirring for 2.25 hours, then poured into saturated $NaHCO_3$ and extracted three times with EtOAc. The combined organic layers were dried over Na_2SO_4 and concentrated *in vacuo*. The resulting crude product was largely starting material (*ca.* 60%), but contained *ca.* 30% 2α-product, accompanied by *ca.* 5% each of the 4α- and 2,2-dialkylated products. A sample was subjected to preparative TLC (2% TEA in EtOAc) to isolate the product.

2α-(1H-Imidazol-2-ylmethyl)-cholestan-3-one: ^{1}H NMR (600 MHz, $CDCl_3$): 6.904 (2H, s), 2.959 (1H, dd, J = 9.5, 14.8 Hz), 2.93-2.77 (2H, m), 2.355 (1H, t, J = 14.2 Hz), 2.148 (1H, dd, J = 5.7, 13.1 Hz), 2.102 (1H, dd, J = 3.6, 14.0 Hz), 1.980 (1H, dt, J = 3.4, 12.7 Hz), 1.054 (3H, s), 0.898 (3H, d, J = 6.6 Hz), 0.865 (3H, d, J = 6.6 Hz), 0.860 (3H, d, J = 6.6 Hz), 0.664 (3H, s).

bioRxiv preprint doi: https://doi.org/10.64898/2026.03.09.710561; this version posted March 11, 2026. The copyright holder for this preprint (which was not certified by peer review) is the author/funder, who has granted bioRxiv a license to display the preprint in perpetuity. It is made available under aCC-BY 4.0 International license.

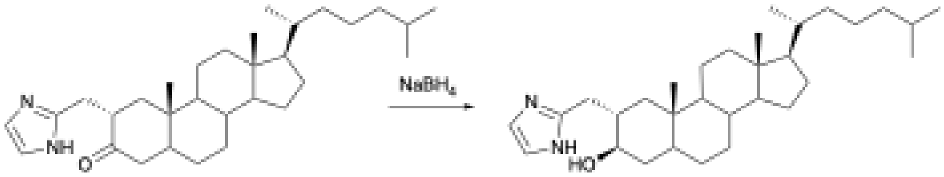

To a solution of imidazole ketone (18.1 mg, 0.039 mmol) in 1.5 ml 4:1 MeOH/DCM, was added $CeCl_3$-$7H_2O$ (24 mg, 1.7 eq.) and the solution was mixed well until the solids had dissolved. An excess of solid $NaBH_4$ was then added (10 mg, 6.7 eq) and the solution was stirred for 1.5 hr. The solvent was evaporated to approx. half the volume with a stream of N_2 and the mixture was extracted with satd. $NaHCO_3$ and EtOAc. The combined organic extracts were dried with Na_2SO_4 and the solvent evaporated with a stream of N_2. Purification was accomplished by preparative TLC (1% TEA in EtOAc/MeOH 19:1).

2α-(1H-Imidazol-2-ylmethyl)-cholestan-3β-ol (2-AMI). ^{1}H NMR (600 MHz, $CDCl_3$): 6.968 (2H, s), 3.358 (1H, td, J = 4.6, 10.6 Hz), 2.91-2.82 (2H, m), 0.897 (3H, d, J = 6.5 Hz), 0.865 (3H, d, J = 6.6 Hz), 0.860 (3H, d, J = 6.6 Hz), 0.803 (3H, s), 0.636 (3H, s). ^{13}C NMR (201 MHz, $CDCl_3$): 147.94, 119.67, 75.33, 56.42, 56.28, 54.18, 44.91, 44.37, 42.56, 39.92, 39.76, 39.51, 38.02, 36.35, 36.16, 35.78, 35.27, 32.10, 31.92, 29.69, 28.22, 28.00, 24.18, 23.84, 22.81, 22.55, 21.25, 18.67, 12.94, 12.07.

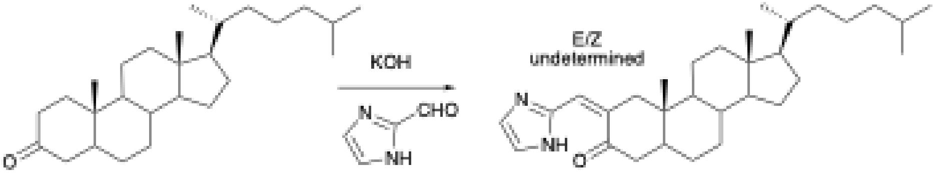

In an alternative synthesis that also provided the 2β-isomer (2-BMI), cholestane-3-one (75.2 mg, 0.19 mmol) in 2.5 ml EtOH was treated with 21 mg powdered KOH (2 eq.) and 22 mg imidazole-2-carbaldehyde (1,2 eq.) at reflux for 5 hr. The mixture was poured into H_2O containing NaCl and extracted with hexanes/EtOAc 1:2. The organic layers were dried over Na_2SO_4 and concentrated *in vacuo*. The product was purified by washing with hexanes.

2-(1H-Imidazol-2-ylmethylene)-cholestan-3β-ol. ^{1}H NMR (600 MHz, $CDCl_3$): 7.388 (2H, s), 7.242 (1H, s), 3.938 (1H, d, J = 17.4 Hz), 2.451 (1H, dd, J = 5.3, 19.0 Hz), 2.29-2.20 (2H, m), 2.06-2.02 (1H, m), 0.919 (3H, d, J = 6.5 Hz), 0.869 (3H, d, J = 6.6 Hz), 0.866 (3H, d, J = 6.6 Hz), 0.827 (3H, s), 0.669 (3H, s).

bioRxiv preprint doi: https://doi.org/10.64898/2026.03.09.710561; this version posted March 11, 2026. The copyright holder for this preprint (which was not certified by peer review) is the author/funder, who has granted bioRxiv a license to display the preprint in perpetuity. It is made available under aCC-BY 4.0 International license.

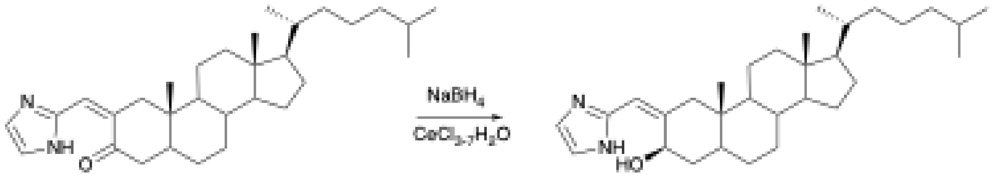

The imidazolylmethylene ketone (4.6 mg, 10 mmol) was dissolved in 1 ml 3:1 MeOH/DCM, an excess of $CeCl_3$-$7H_2O$ (11.1 mg, 3 eq.) was added and stirred for 5 min., followed by addition of $NaBH_4$ (excess). After 20 min., the solvent was evaporated to approx. half the volume with a stream of N_2 and a few drops of 10% HCl were added to destroy the excess $NaBH_4$. The mixture was extracted with half-saturated $NaHCO_3$ and EtOAc, dried with Na_2SO_4, and the solvent was evaporated with a stream of N_2.

2-(1H-Imidazol-2-ylmethylene)-cholestan-3β-ol. ^{1}H NMR (600 MHz, $CDCl_3$): 7.069 (2H, s), 6.465 (1H, s), 4.18-4.13 (1H, m), 4.18-4.13 (1H, m), 3.500 (1H, d, J = 13.1 Hz), 0.893 (3H, d, J = 6.4 Hz), 0.861 (3H, d, J = 6.6 Hz), 0.857 (3H, d, J = 6.6 Hz), 0.609 (3H, s), 0.605 (3H, s).

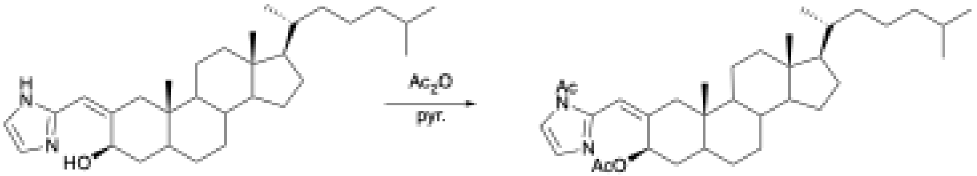

The imidazolylmethylene sterol was acetylated using 3 drops of Ac_2O in 0.3 mL pyridine at 40 ˚C, ON. Evaporation of the volatiles with a stream of N_2 gave a product acetylated at both the 3-position and the imidazole ring.

N-Acetyl-2-(Imidazol-2-ylmethylene)-cholestan-3β-yl acetate. ^{1}H NMR (600 MHz, $CDCl_3$): 7.241 (1H, d, J = 1.5 Hz), 7.018 (1H, d, J = 1.5 Hz), 6.823 (1H, s), 5.347 (1H, dd, J = 5.4, 11.0 Hz), 3.859 (1H, d, J = 13.3 Hz), 2.565 (3H, s), 2.177 (3H, s), 0.891 (3H, d, J = 6.4 Hz), 0.860 (3H, d, J = 6.6 Hz), 0.855 (3H, d, J = 6.6 Hz), 0.716 (3H, s), 0.626 (3H, s).

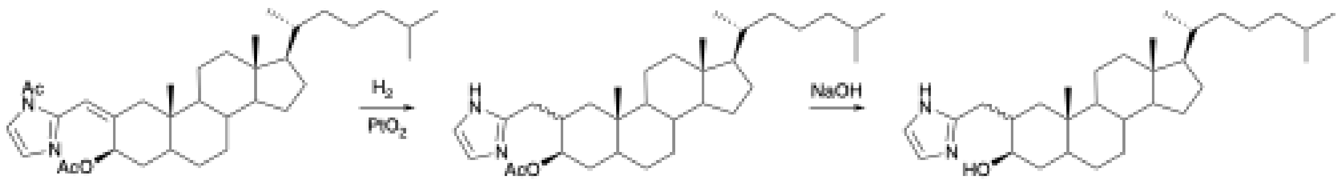

The protected imidazolylmethylene sterol (5 mg) dissolved in 1.25 mL of EtOH was hydrogenated in the presence of 1.5 mg of Adam’s catalyst for 4 hours with stirring, followed by filtration with celite. The product consisted of 63% 2β- and 37% 2α-isomers. Preparative TLC (2% TEA in hexanes/EtOAc 3:2) using a TLC plate pretreated with 2% TEA in hexanes was used to remove the recovered starting material

bioRxiv preprint doi: https://doi.org/10.64898/2026.03.09.710561; this version posted March 11, 2026. The copyright holder for this preprint (which was not certified by peer review) is the author/funder, who has granted bioRxiv a license to display the preprint in perpetuity. It is made available under aCC-BY 4.0 International license.

(20%). The mixture of hydrogenated products (2-3 mg) in 0.2 mL DCM, was saponified with 0.5 mL of 10% NaOH/MeOH with stirring for 1 hr. The reaction was diluted with 7 mL of half-saturated $NaHCO_3$ and extracted repeatedly with EtOAc. The organic layers were dried with Na_2SO_4 and the product purified by preparative TLC (2% TEA in hexanes/EtOAc 1:2) using a TLC plate pretreated with 2% TEA in hexanes to separate the 2α- and 2β diastereomers.

2β-(1H-Imidazol-2-ylmethyl)-cholestan-3β-ol (2-BMI). ^{1}H NMR (600 MHz, $CDCl_3$): 6.891 (2H, s), 3.953 (1H, tt, J = 5.1,5.8 Hz), 3.307 (1H, dd, J = 9.0,15.6 Hz), 2.734 (1H, d, J = 15.6 Hz), 2.57-2.52 (1H, m), 0.892 (3H, d, J = 6.5 Hz), 0.889 (3H, s), 0.861 (3H, d, J = 6.6 Hz), 0.857 (3H, d, J = 6.6 Hz), 0.646 (3H, s). ^{13}C NMR (201 MHz, $CDCl_3$): 150.44, 120.4 (detected by HSQC), 72.84, 56.39, 56.29, 55.25, 45.84, 44.42, 42.66, 40.07, 39.51, 39.05, 36.16, 35.92, 35.79, 35.06, 33.93, 31.95, 30.58, 28.26, 28.23, 28.00, 24.16, 23.83, 22.80, 22.55, 21.30, 18.64, 14.91, 12.14.

Synthesis of 3β-aminoxy-5β-cholestane (2-BMI):

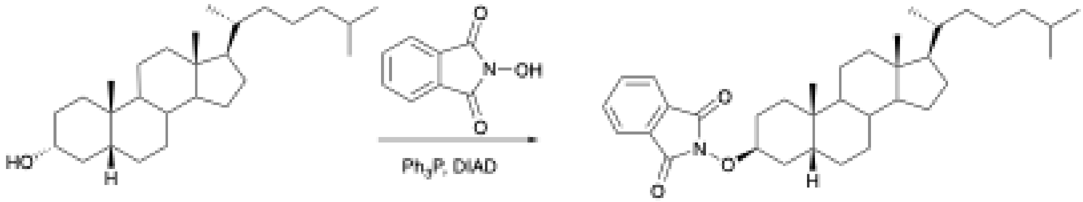

To a solution of 5β-cholestan-3α-ol (17.0 mg, 43.8 µmol) dissolved in 0.5 mL of anhydrous THF under a N_2 atmosphere was added triphenylphosphine (20.5 mg, 2.0 eq) and *N*-hydroxyphthalimide (12.6 mg, 2.0 eq) and the contents were mixed well to dissolve, followed by the addition of DIAD (15 µL, 2.0 eq). The mixture was allowed to stand at rt for 5.5 hours, then quenched with 5 mL of satd. $NaHCO_3$ and extracted thoroughly with hexanes/EtOAc 9:1. The combined organic extracts were filtered through silica which was washed with hexanes/EtOAc 2:1 to ensure complete elution. The product were purified by preparative TLC (hexanes/EtOAc 9:1) to afford the product with inversion of the stereochemistry at C-3 in 90% yield (99% brsm).

bioRxiv preprint doi: https://doi.org/10.64898/2026.03.09.710561; this version posted March 11, 2026. The copyright holder for this preprint (which was not certified by peer review) is the author/funder, who has granted bioRxiv a license to display the preprint in perpetuity. It is made available under aCC-BY 4.0 International license.

3β-Phthalimidooxy-5β-cholestane. ^{1}H NMR (600 MHz, $CDCl_3$): 7.818 (2H, dd, J = 3.1, 5.3 Hz), 7.729 (2H, dd, J = 3.1, 5.3 Hz), 4.506 (1H, m), 1.035 (3H, s), 0.899 (3H, d, J = 6.5 Hz), 0.863 (3H, d, J = 6.6 Hz), 0.858 (3H, d, J = 6.6 Hz), 0.659 (3H, s). ^{13}C NMR (151 MHz, $CDCl_3$): 164.31, 134.29, 129.14, 123.35, 84.02, 56.69, 56.43, 42.76, 40.30, 40.03, 39.52, 36.69, 36.19, 35.77, 35.68, 34.87, 30.09, 29.83, 28.31, 28.00, 26.42, 26.11, 24.22, 23.82, 23.74, 22.78, 22.54, 21.13, 18.69, 12.06.

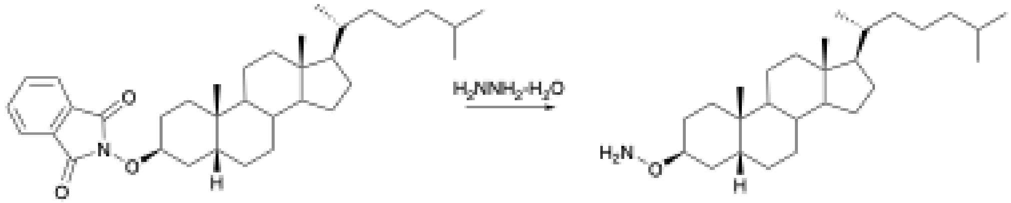

To a solution of the phthalimidooxy intermediate (5.0 mg, 9.4 μmol) in 0.5 mL of anhydrous DCM was added 1 drop (approx. 50 μL) of hydrazine hydrate under a N_2 atmosphere. The solutions were mixed well and allowed to stand at rt for 2.25 hours. The contents were then filtered through celite and concentrated *in vacuo* at rt to afford the desired product in quantitative yield.

3β-Aminoxy-5β-cholestane (2-BMI). ^{1}H NMR (600 MHz, $CDCl_3$): 5.165 (2H, br s), 3.790 (1H, m), 1.977 (1H, dt, J = 3.5, 12.5 Hz), 1.90-0.95 (m), 0.929 (3H, s), 0.901 (3H, d, J = 6.5 Hz), 0.866 (3H, d, J = 6.6 Hz), 0.861 (3H, d, J = 6.6 Hz), 0.647 (3H, s). ^{13}C NMR (151 MHz, $CDCl_3$): 79.15, 56.71, 56.44, 42.76, 40.33, 39.95, 39.54, 37.17, 36.21, 35.80, 35.72, 34.97, 30.48, 29.77, 28.33, 28.02, 26.75, 26.22, 24.24, 23.86, 23.85, 23.69, 22.80, 22.56, 21.12, 18.70, 12.07.

Synthesis of 24-azido-5α-chol-5-en-3β-ol:

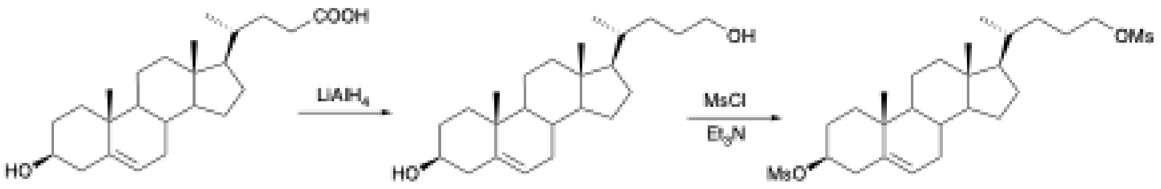

To a solution of 5-cholenic acid (50.3 mg, 0.13 mmol) in 20 mL of anhydrous THF was added solid LAH (62.5 mg, 1.6 mmol, 13 eq.). The mixture was refluxed overnight, then cooled to rt, and excess LAH was destroyed by careful addition of 2 mL of 10% HCl. The mixture was then filtered and concentrated, excess water was removed by azeotrope with benzene. The residue was taken up in EtOAc, filtered through

bioRxiv preprint doi: https://doi.org/10.64898/2026.03.09.710561; this version posted March 11, 2026. The copyright holder for this preprint (which was not certified by peer review) is the author/funder, who has granted bioRxiv a license to display the preprint in perpetuity. It is made available under aCC-BY 4.0 International license.

Na_2SO_4, then concentrated to dryness under a stream of N_2. The crude product was dissolved in 4 mL of anhydrous DCM and 0.1 mL of anhydrous TEA (0.72 mmol), followed by 50 µL of MsCl (0.65 mmol). After 1 hour, complete conversion was observed by TLC and the reaction was quenched with 3.5 mL of 10% HCl and extracted several times with hexanes/EtOAc 2:1. The combined organic extracts were washed with satd. $NaHCO_3$, dried over Na_2SO_4 and concentrated. Purification by silica gel column chromatography (hexanes/EtOAc 2:1) gave the dimesylate (39.4 mg, 59%).

5α-Chol-5-ene-3β,24-dimesylate ester. ^{1}H NMR (600 MHz, $CDCl_3$): 5.418 (1H, m), 4.521 (1H, m), 4.24-4.16 (2H, m), 3.005 (3H, s), 3.000 (3H, s), 1.017 (3H, s), 0.939 (3H, d, *J* = 6.5 Hz), 0.680 (3H, s).

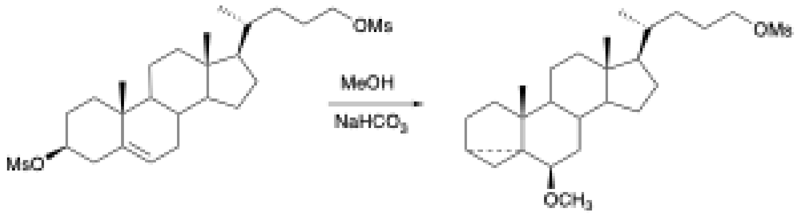

A solution of the dimesylate (19.7 mg, 0.038 mmol) was treated with saturated $NaHCO_3$ (350 µL) in methanol (7 mL) at reflux for 1.5 hours. The mixture was diluted with water (40 mL), extracted several times with hexanes/EtOAc 4:1, dried over Na_2SO_4 and concentrated. The crude product was purified by preparative TLC (hexanes/EtOAc 4:1) to give 10.2 mg of the i-methyl ether (59%), containing about 9% of the normal methyl ether which was not separated.

24-Methanesulfonoxy-5α-cholane i-methyl ether: ^{1}H NMR (600 MHz, $CDCl_3$). 4.24-4.16 (2H, m), 3.320 (3H, s), 3.000 (3H, s), 2.768 (1H, t, *J* = 2.6 Hz), 1.019 (3H, s), 0.935 (3H, d, *J* = 6.5 Hz), 0.718 (3H, s).

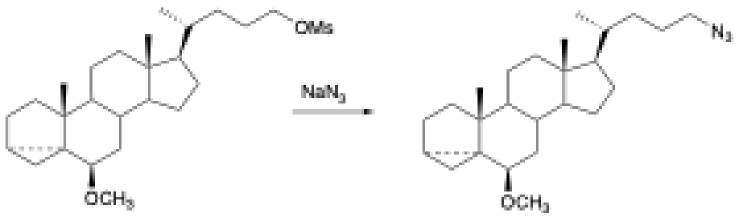

To a solution of the i-methyl mesylate (9.2 mg, 0.02 mmol) in 0.7 mL of anhydrous DMF under N_2, was added NaN_3 (7.2 mg, 0.11 mmol). The mixture was stirred at 75 °C for 2 hr, at which point TLC showed complete conversion. The contents were diluted with 7 ml half-saturated $NaHCO_3$, extracted thoroughly with hexanes/EtOAc 2:1 and dried over Na_2SO_4. Concentration gave 8.0 mg of product (98%) which was used without further purification.

24-Azido-5α-cholane i-methyl ether: ^{1}H NMR (600 MHz, $CDCl_3$). 3.322 (3H, s), 3.26-3.18 (2H, m), 2.769 (1H, t, *J* = 2.7 Hz), 1.020 (3H, s), 0.933 (3H, d, *J* = 6.5 Hz), 0.719 (3H, s).

bioRxiv preprint doi: https://doi.org/10.64898/2026.03.09.710561; this version posted March 11, 2026. The copyright holder for this preprint (which was not certified by peer review) is the author/funder, who has granted bioRxiv a license to display the preprint in perpetuity. It is made available under aCC-BY 4.0 International license.

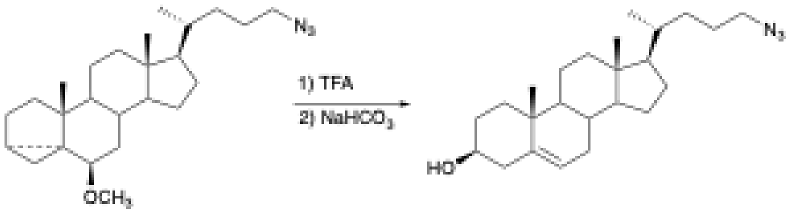

The azido i-methyl ether (7.2 mg, 0.018 mmol) was deprotected in 0.65 mL of DCM containing 1% TFA. When the reaction was found to be complete by TLC, the solution was concentrated to dryness under a stream of N_2 with gentle heating, and the residue was dissolved in 1 mL of MeOH/DCM 4:1. Three drops of sat. $NaHCO_3$ were added and the solution mixed well. The TLC spot at R_f = 0.7 was replaced by one at R_f = 0.2 (hexanes/EtOAc 4:1) after 20 min. After partitioning between water and hexanes/EtOAc 2:1, the sample was dried with Na_2SO_4 and purified by preparative TLC (hexanes/EtOAc 4:1) to give 2.7 mg (38% yield).

24-Azido-5α-chol-5-en-3β-ol: ^{1}H NMR (600 MHz, $CDCl_3$). 5.350 (1H, m), 3.523 (1H, tt, *J* = 4.5, 11.3 Hz), 3.28-3.18 (2H, m), 1.006 (3H, s), 0.940 (3H, d, *J* = 6.5 Hz), 0.682 (3H, s).

1. Owen, T. S.; Ngoje, G.; Lageman, T. J.; Bordeau, B. M.; Belfort, M.; Callahan, B. P., Forster resonance energy transfer-based cholesterolysis assay identifies a novel hedgehog inhibitor. *Anal Biochem* **2015,** *488*, 1-5.
2. Owen, T. S.; Xie, X. J.; Laraway, B.; Ngoje, G.; Wang, C.; Callahan, B. P., Active site targeting of hedgehog precursor protein with phenylarsine oxide. *Chembiochem* **2015,** *16* (1), 55-8.
3. Smith, C. J.; Wagner, A. G.; Stagnitta, R. T.; Xu, Z.; Pezzullo, J. L.; Giner, J. L.; Xie, J.; Covey, D. F.; Wang, C.; Callahan, B. P., Subverting Hedgehog Protein Autoprocessing by Chemical Induction of Paracatalysis. *Biochemistry* **2020,** *59* (6), 736-741.
4. Wagner, A. G.; Stagnitta, R. T.; Xu, Z.; Pezzullo, J. L.; Kandel, N.; Giner, J. L.; Covey, D. F.; Wang, C.; Callahan, B. P., Nanomolar, Noncovalent Antagonism of Hedgehog Cholesterolysis: Exception to the "Irreversibility Rule" for Protein Autoprocessing Inhibition. *Biochemistry* **2022,** *61* (11), 1022-1028.
5. Childs, M. E.; Weber, W. P., Preparation of Cyanoformates - Crown Ether Phase Transfer Catalysis. *J Org Chem* **1976,** *41* (21), 3486-3487.
